# Integrative machine learning models reveal immune and metabolic signatures predictive of colorectal cancer prognosis

**DOI:** 10.1007/s12672-026-04758-y

**Published:** 2026-03-03

**Authors:** Zhanyuan Sun, Zijing Wang, Qingchen Lv, Shaohua Hou, Guo Li, Tao Jiang, Hai Li

**Affiliations:** 1 First Clinical Medical College, General Hospital of Ningxia Medical University, 804 Shengli Road, Yinchuan, 750004 China; 2 Ningxia Key Laboratory of Stem Cell and Regenerative Medicine, Institute of Medical Sciences, General Hospital of Ningxia Medical University, Yinchuan, 750004 Ningxia Hui Autonomous Region China; 3 Department of Pediatrics, Nantong First People’s Hospital (The Second Affiliated Hospital of Nantong University), Nantong, 226001 Jiangsu China; 4 Department of Anal-Colorectal Surgery, General Hospital of Ningxia Medical University, 804 Shengli Road, Yinchuan, 750004 China

**Keywords:** Colorectal cancer, Immune and metabolic-related genes, Prognosis, Machine learning, IL20RB

## Abstract

**Background:**

The progression of colorectal cancer (CRC) is profoundly shaped by immune and metabolic dysregulation within the tumor microenvironment (TME). This study aimed to develop an immune–metabolism-related gene (IMRG)-based prognostic model and experimentally validate its key molecular drivers.

**Methods:**

Transcriptomic and clinical data from the TCGA and GEO cohorts were analyzed to identify differentially expressed IMRGs. Molecular subtypes were defined using non-negative matrix factorization (NMF) clustering, and a machine learning–based prognostic model integrating 101 algorithmic combinations was constructed and validated. Gene Set Enrichment Analysis (GSEA), immune infiltration profiling, and drug sensitivity analyses were performed to elucidate biological and immunological differences. IL20RB, identified as a hub IMRG, was further validated through qRT–PCR, Western blotting, and IHC. Its functional role was assessed using CCK-8, wound-healing, and Transwell assays.

**Results:**

NMF clustering revealed two distinct molecular subtypes of CRC with divergent survival outcomes and immune characteristics. The 4-gene IMRG-based model demonstrated robust prognostic performance (C-index = 0.657) and was externally validated in a GEO cohort (AUC up to 0.824 for 1-, 3-, and 5-year survival). GSEA showed that the high-risk group was enriched for complement/coagulation cascades and extracellular matrix remodeling, whereas the low-risk group was enriched for metabolic programs including butanoate metabolism and the TCA cycle. Immune profiling indicated increased CD8⁺ T-cell infiltration in the high-risk group and a distinct immune checkpoint landscape, with most checkpoints elevated in the low-risk group, while ADORA2A, TNFRSF25 and CD276 were relatively higher in the high-risk group. Experimental analyses confirmed that IL20RB was significantly upregulated in CRC tissues and cell lines, and its silencing markedly inhibited CRC cell proliferation, migration, and invasion, consistent with its predicted oncogenic role.

**Conclusion:**

This integrative transcriptomic and computational analysis combined with machine learning and experimental validation identified IMRGs—particularly IL20RB—as critical mediators of CRC progression. The proposed IMRG-based prognostic model offers a robust framework for patient risk stratification, immune landscape characterization, and therapeutic targeting in CRC.

**Supplementary Information:**

The online version contains supplementary material available at 10.1007/s12672-026-04758-y.

## Introduction

Colorectal cancer (CRC) remains one of the most prevalent and lethal malignancies worldwide [1–3], accounting for approximately 10.9% of cancer cases in men and 9.5% in women [3]. Despite significant progress in early detection and the emergence of novel therapeutic modalities such as immunotherapy and targeted treatments, the five-year survival rate for advanced-stage CRC remains unsatisfactory [4]. In 2020 alone, there were an estimated 1.9 million new CRC cases and nearly 935,000 deaths globally, underscoring the substantial public health burden of this disease [5, 6]. Clinical evidence indicates that immune checkpoint inhibitors have demonstrated therapeutic efficacy in metastatic CRC patients exhibiting high microsatellite instability (MSI-H) or defective mismatch repair (dMMR) [7]. However, due to the intricate and heterogeneous nature of the tumor microenvironment (TME), only a subset of patients benefit significantly from these treatments [8, 9]. This limitation highlights the pressing need to identify more precise biomarkers and develop advanced therapeutic strategies to improve prognostic accuracy and treatment outcomes in CRC.

Emerging evidence has revealed that dysregulation of metabolic pathways plays a pivotal role in tumor immune evasion [10], profoundly affecting immune cell function within the TME. A hallmark example is the Warburg effect [11], in which tumor cells preferentially engage in glycolysis over oxidative phosphorylation, even under normoxic conditions [12, 13]. This metabolic reprogramming not only sustains rapid tumor proliferation but also results in lactate accumulation, which suppresses T cell proliferation and cytotoxic activity, thereby weakening anti-tumor immunity. Furthermore, aberrant lipid metabolism in tumor cells generates immunosuppressive lipid metabolites that impair macrophage-mediated tumor clearance and promote M2 macrophage polarization, facilitating tumor progression and metastasis [14]. Alterations in amino acid metabolism—particularly glutamine dependence—further sustain tumor growth while diminishing the effector functions of T cells and natural killer (NK) cells [15]. Additionally, tumor cells frequently overexpress key metabolic enzymes such as indoleamine 2,3-dioxygenase (IDO), which catalyzes tryptophan degradation into immunosuppressive metabolites, contributing to T cell exhaustion and immune escape [16]. Metabolic byproducts, including adenosine and lactate, also interact with immune checkpoint pathways such as PD-1/PD-L1, amplifying immune suppression and attenuating anti-tumor responses [17]. Collectively, these findings highlight the intricate crosstalk between tumor metabolism and immune regulation, suggesting that targeting metabolic pathways may enhance the efficacy of cancer immunotherapy. A comprehensive understanding of these metabolic alterations and their immunological implications is therefore crucial for the development of innovative therapeutic interventions.

In this study, we applied machine learning approaches to construct and validate a prognostic risk model for CRC based on immune–metabolism-related genes (IMRGs). The model was systematically evaluated for its predictive performance and clinical utility in survival stratification, immune landscape profiling, and therapeutic response prediction. Among the identified IMRGs, IL20RB emerged as a key gene strongly associated with CRC prognosis. Its expression patterns and biological functions were further validated through molecular and cellular experiments. Collectively, this integrative study combines computational modeling with experimental validation to refine prognostic assessment and identify novel therapeutic targets in CRC.

## Materials and methods

### Data acquisition and processing

RNA sequencing data and corresponding clinical information for CRC patients—including 51 normal and 650 tumor samples—were obtained from The Cancer Genome Atlas (TCGA) database (http://cancergenome.nih.gov/). IMRGs were defined as the union of ImmPort immune-related genes (IRGs; http://www.immport.org/) and MSigDB KEGG metabolism-related genes (MRGs; C2:CP: KEGG; https://www.gsea-msigdb.org/), with duplicated gene symbols removed. The full IMRG list and database access details are provided in Supplementary Table S1. In addition, the GSE39582 dataset from the Gene Expression Omnibus (GEO) database (https://www.ncbi.nlm.nih.gov/geo/)—which contains comprehensive gene expression profiles and corresponding clinical data—was used for external validation of the model. To minimize bias across datasets and ensure data consistency, transcriptomic data were normalized using the *limma* R package with log2 transformation [log2(x + 1)]. To further correct for batch effects between the TCGA and GEO cohorts, the ComBat function implemented in the *sva* R package was applied. Principal component analysis (PCA) conducted before and after correction confirmed the effective elimination of batch effects (Figure S1), thereby ensuring the reliability and comparability of downstream analyses.

### Screening for differentially expressed IMRGs

Expression profiles of IMRGs were preprocessed using the *limma* R package. Genes with an average expression level below 0.5 were removed to ensure inclusion of only adequately expressed IMRGs for subsequent analyses. Differences in gene expression between normal and tumor tissues were evaluated using the Wilcoxon rank-sum test. Differentially expressed genes (DEGs) were defined by a |log₂ fold change (logFC)| > 1 and an adjusted *P* < 0.05. To illustrate gene expression patterns, the 50 most significantly upregulated and 50 most significantly downregulated IMRGs, ranked by logFC, were visualized. A heatmap was generated using the *pheatmap* R package, while a volcano plot depicting both statistical significance and expression magnitude was produced using *ggplot2*.

### Non-negative matrix factorization (NMF) clustering Analysis

To identify potential molecular subtypes based on IMRG expression, transcriptomic profiles and survival data for the differentially expressed IMRGs from the TCGA and GEO cohorts were extracted, preprocessed, and merged following batch correction. NMF clustering was then performed using the *“brunet”* algorithm with 50 iterations (*nrun* = 50) to ensure clustering stability. A range of cluster numbers (*k* = 2–10) was evaluated, and multiple statistical indices—including the cophenetic correlation coefficient, dispersion, silhouette width, residual sum of squares (RSS), explained variance, and sparseness—were used to assess clustering performance. Based on the integrated evaluation of these metrics, *k* = 2 was selected as the optimal number of clusters. Consensus heatmaps and PCA plots were subsequently generated to verify the robustness and visual separability of the identified subtypes.

To explore subtype-specific biological characteristics, gene expression differences were visualized using heatmaps. Kaplan–Meier (K–M) survival analyses were conducted to compare overall survival (OS) and progression-free survival (PFS) between the two subtypes. TME characteristics were further evaluated using the ESTIMATE algorithm implemented in the estimate R package, which infers immune and stromal cell content from bulk expression data. In addition to ImmuneScore, StromalScore, and ESTIMATEScore, tumor purity was estimated using the ESTIMATE algorithm and compared between subtypes. Violin plots generated with ggviolin depicted the distributions of ImmuneScore, StromalScore, and ESTIMATEScore across subtypes. A separate violin plot was generated to visualize tumor purity differences between C1 and C2. In addition, immune cell infiltration was quantified using R packages including plyr, reshape2, ggplot2, and ggpubr. Boxplots created with ggboxplot illustrated differences in immune cell abundance among subtypes. A comprehensive heatmap was finally constructed to summarize TME-related characteristics and immune infiltration profiles across the identified molecular subtypes.

To further annotate the NMF subtypes, we assessed their associations with established CRC molecular classifications. Consensus molecular subtypes (CMS, CMS1–CMS4) were inferred using the CMScaller method based on bulk transcriptomic profiles, and the CMS composition between C1 and C2 was compared using Fisher’s exact test. MSI status (MSI-H, MSI-L, MSS) was obtained from the corresponding dataset and dichotomized as MSI-H (dMMR) versus MSI-L/MSS (pMMR); differences between clusters were assessed using Fisher’s exact test. Finally, to address potential confounding due to stromal/immune content and tumor purity, we performed a purity-adjusted Cox regression model including Cluster and tumor purity as covariates.

### Construction and validation of prognostic risk models

After obtaining the training dataset from the TCGA cohort, univariate Cox regression analysis was performed to identify IMRGs significantly associated with CRC prognosis (*P* < 0.05). The resulting prognostic IMRGs were subsequently used to construct survival prediction models within a machine learning–based ensemble framework. A total of 101 combinations of machine learning algorithms were evaluated, including individual learners (e.g., random survival forest [RSF], elastic net/LASSO, CoxBoost, survival-SVM, SuperPC, and GBM) and hybrid pipelines combining feature selection and survival modeling (e.g., RSF + plsRcox, LASSO + GBM). To ensure comparable feature spaces across cohorts, genes shared by TCGA and GEO were intersected, and expression matrices were standardized (centering and scaling) using scaleData (centerFlags = TRUE, scaleFlags = TRUE). A fixed random seed (set.seed = 123) was applied for reproducibility, and models with ≤ 5 selected variables were excluded to avoid instability (min.selected.var = 5).

Within the TCGA cohort, algorithm-specific internal cross-validation was used for hyperparameter/threshold selection when applicable (e.g., 10-fold CV in cv.glmnet for elastic net/LASSO, K = 10 in cv.CoxBoost to determine the optimal step number, 10-fold CV in superpc.cv to select the optimal threshold, cv.plsRcox to select the number of components, and cv.folds = 10 in GBM to determine the optimal number of trees). Models were then fitted in TCGA using the selected settings and evaluated by Harrell’s concordance index (C-index). The GEO cohort (GSE39582) was used as an independent external validation dataset. C-index values were calculated in both TCGA and GEO based on a univariate Cox model of survival against the predicted risk score (RS) within each cohort.

Among all tested combinations, the RSF + plsRcox model achieved the best overall performance and was selected as the final prognostic model. In this hybrid pipeline, RSF was used for feature selection, and the final RS was generated by the plsRcox model as a linear predictor. Specifically, RS was calculated as: RS = Σ(β_i_ × Expr_i_), where β_i_ represents the plsRcox coefficient and Expr_i_ denotes the standardized expression level of each selected gene. Patients were stratified into high-risk and low-risk groups using the cohort-specific median RS (i.e., the median RS within each cohort). For visualization across datasets, RS values could be additionally z-score standardized; however, this transformation was not used to define the risk-group cutoff. Model performance was evaluated using Kaplan–Meier survival analysis, time-dependent ROC curves, and multivariate Cox regression. A nomogram (rms package) was employed to visualize individualized survival probabilities. Model accuracy was further assessed using calibration curves and C-index values, while correlations between RS and clinical characteristics were illustrated with boxplots.

### Gene set enrichment and drug sensitivity analyses

To explore transcriptional differences and biological pathway alterations between tumor samples from distinct risk groups, log-transformed gene expression ratios were first computed and organized. Gene Set Enrichment Analysis (GSEA) was performed using the *clusterProfiler* R package to identify significantly enriched KEGG pathways associated with either the high- or low-risk cohorts. Pathways with *P* < 0.05 were considered significant, and the top five enriched pathways in each group were visualized using the *enrichplot* package.

Drug sensitivity analysis was conducted to evaluate potential therapeutic differences between risk groups. The half maximal inhibitory concentration (IC₅₀) values of various chemotherapeutic agents for TCGA patients were estimated using the *oncoPredict* R package. Differences in IC₅₀ values between high- and low-risk groups were assessed using the Wilcoxon rank-sum test, and the results were visualized with boxplots.

### Assessment of tumor-infiltrating immune cells for prognostic modeling and immunotherapy response evaluation

The infiltration of immune cell populations in tumor samples was quantified using the *MCPcounter* R package [18]. To evaluate differences in immune cell abundance between risk groups, cyclic analyses were performed for each immune cell type. In each iteration, the distribution of immune cell levels in high- and low-risk cohorts was visualized using violin plots generated with the *ggviolin* function, while statistical comparisons between groups were conducted using the *stat_compare_means* function. A correlation matrix of immune cell infiltration scores was then constructed and visualized using the *corrplot* package to illustrate intercellular relationships and potential co-infiltration patterns. Furthermore, the association between IMRG-derived risk features and predicted immunotherapy response was analyzed using data from the Cancer Immunome Atlas (TCIA) database (https://tcia.at/home). This analysis enabled the evaluation of potential correlations between the IMRG-based prognostic model and the likelihood of response to immune checkpoint blockade therapies.

### Comparison of different risk models

To evaluate the prognostic performance of various gene signature–based risk models, each signature dataset was analyzed in a cyclic processing framework. RS for each signature were calculated using the Cox proportional hazards model, and these scores were subsequently integrated with survival data to predict individual sample risk values via the *predict* function. K–M survival analyses were performed to compare survival outcomes between high- and low-risk groups. To assess the time-dependent predictive performance of each model, receiver operating characteristic (ROC) curves and corresponding area under the curve (AUC) values were generated at 1-, 3-, and 5-year intervals using the *timeROC* R package. In addition, the C-index for each model was calculated to quantify predictive accuracy. Comparative results were visualized using bar plots, enabling a direct comparison of the prognostic efficacy among different gene signatures.

### Clinical sample collection

This study collected clinical tissue specimens from 30 CRC patients who underwent curative surgical resection at the General Hospital of Ningxia Medical University. All samples were obtained from the same patient cohort (Supplementary Table S2). For qRT-PCR analysis, 30 paired CRC tissues and the corresponding adjacent normal intestinal epithelial tissues were analyzed. For WB, 12 representative paired tissue samples were selected from the above cohort. IHC was performed on all 30 paired formalin-fixed, paraffin-embedded (FFPE) tissue sections. All enrolled patients had no severe comorbidities and had not received preoperative radiotherapy or chemotherapy. Written informed consent was obtained from all participants and their family members prior to sample collection. The study protocol was reviewed and approved by the Ethics Committee of the General Hospital of Ningxia Medical University (approval no. KYLL-2024-1400).

### Cell culture and siRNA transfection

This study utilized one human normal colon epithelial cell line (NCM460) and four human CRC cell lines (HT29, HCT116, SW480, and SW620), all obtained from the American Type Culture Collection (ATCC). IL20RB gene silencing was performed in the HT29 and SW620 CRC cell lines using specific small interfering RNAs (siRNAs) synthesized by Sangon Biotech (Shanghai, China). One day prior to transfection, cells were seeded at an appropriate density in 6-well plates. During transfection, siRNA (final concentration: 50 nM) and transfection reagent were each diluted in Opti-MEM serum-free medium and incubated at room temperature for 15 min to allow complex formation. The resulting complexes were then added dropwise to the cell culture medium. After 6–8 h of incubation, the medium was replaced with complete growth medium, and the cells were cultured continuously. Cells were harvested at 48 and 72 h post-transfection, and IL20RB knockdown efficiency at both the mRNA and protein levels was evaluated using qRT-PCR and WB, respectively.

### Immunohistochemistry (IHC)

Paraffin-embedded tissue Sect. (4 μm thick) were baked in a 70 °C oven for 20 min, followed by deparaffinization in xylene for 10 min (repeated three times). The sections were then rehydrated through a graded ethanol series (100%, 95%, and 75%) for 5 min each. Antigen retrieval was performed using the high-pressure heat method to expose antigenic epitopes. After cooling to room temperature, the sections were incubated with the primary antibody overnight at 4 °C. The following day, a secondary antibody was applied, and incubation was continued for 30 min at 37 °C. Color development was achieved using 3,3′-diaminobenzidine (DAB) substrate, with the reaction time carefully monitored under a microscope to ensure optimal staining intensity. Sections were then counterstained with hematoxylin, dehydrated through graded alcohols, cleared in xylene, and sealed with neutral balsam. Finally, stained sections were examined and photographed under a light microscope for analysis. Immunostaining was independently evaluated by two experienced pathologists blinded to all clinical information. Discrepancies were resolved by joint review to reach a consensus. IL20RB expression was semi-quantified using an H-score–like system by multiplying the staining extent score (0, 0%; 1, 1–25%; 2, 26–50%; 3, 51–75%; 4, > 75% positive tumor cells) by the staining intensity score (0, negative; 1, weak; 2, moderate; 3, strong), yielding a final IHC score ranging from 0 to 12.

### Western blotting (WB)

Cell and tissue samples were lysed in lysis buffer supplemented with protease inhibitors, phosphatase inhibitors, and phenylmethylsulfonyl fluoride (PMSF), followed by incubation on ice for 30 min. After centrifugation, the supernatant was collected, and protein concentration was determined using the bicinchoninic acid (BCA) assay. Samples were then diluted to the desired concentration, mixed with loading buffer, and boiled for 5 min to denature proteins prior to storage. Equal amounts of protein were loaded into each lane for SDS–polyacrylamide gel electrophoresis (SDS-PAGE), followed by electrotransfer onto polyvinylidene difluoride (PVDF) membranes. Membranes were blocked with 5% skim milk in TBST (Tris-buffered saline with 0.1% Tween-20) for 2 h at room temperature to prevent non-specific binding. After blocking, membranes were washed three times with TBST (10 min per wash) and incubated overnight at 4 °C with primary antibodies specific to the target proteins. The next day, membranes were washed three additional times and then incubated for 1 h at room temperature with horseradish peroxidase (HRP)-conjugated secondary antibodies corresponding to the primary antibody species. Protein bands were visualized using an enhanced chemiluminescence (ECL) detection system, and band intensities were quantified using ImageJ software. Quantified data were subsequently subjected to statistical analysis. All antibodies used in our study are listed in Supplementary Table S3.

### Quantitative real-time PCR (qRT-PCR)

For RNA extraction, 1 mL of TRIzol reagent was added to each homogenized sample and transferred to a 1.5 mL Eppendorf (EP) tube. Subsequently, 200 µL of chloroform was added, and the samples were vortexed and centrifuged at 15,000 rpm for 15 min at 4 °C. The upper aqueous phase was carefully transferred to a new EP tube, followed by the addition of 200 µL of isopropanol. The mixture was centrifuged at 12,000 rpm for 10 min at 4 °C to precipitate RNA. The resulting RNA pellet was washed twice with 75% ethanol (centrifuged at 12,000 rpm for 5 min at 4 °C each time), air-dried until translucent, and then dissolved in nuclease-free water (ddH₂O). RNA concentration and purity were measured spectrophotometrically. Reverse transcription and qRT–PCR were conducted using commercial kits from Vazyme (Batch Nos. R302-01 and Q411-02; Nanjing, China), following the manufacturer’s protocols. Relative gene expression levels were calculated using the 2^−ΔΔCT^ method, and specific primer sequences are listed in Supplementary Table S4.

### Cell counting kit-8 (CCK-8) assay

Cells were seeded into 96-well plates at a density of 5 × 10³ cells per well and allowed to adhere overnight. At 24, 48, 72, and 96 h, 100 µL of 10% CCK-8 reagent was added to each well, followed by incubation at 37 °C in the dark for 1 h. Cell proliferation was quantified by measuring the absorbance at 450 nm using a microplate reader, and the results were used to generate a cell growth curve.

### Wound-healing assay

Cells were seeded into 6-well plates at a density of 1 × 10⁵ cells/mL and cultured at 37 °C in a 5% CO₂ incubator until a confluent monolayer was formed. The cell layer was washed twice with phosphate-buffered saline (PBS), and a straight scratch was created at the center of each well using a sterile 200 µL pipette tip. Detached cells were gently removed by washing with PBS, and the initial wound area was photographed at 0 h. The plates were then returned to the incubator for continued culture, and images of the same wound sites were captured again at 24 h for migration analysis.

### Transwell assay

A serum-free cell suspension containing 5 × 10⁴ cells in 100 µL was seeded into the upper chamber of the Transwell insert. The lower chamber was filled with 400 µL of culture medium supplemented with 20% fetal bovine serum (FBS) to serve as a chemoattractant. After incubation for 48 h, non-migrated cells on the upper surface of the membrane were gently removed using a cotton swab. Cells that had migrated to the lower surface were fixed with 4% paraformaldehyde for 20 min and stained with 0.1% crystal violet for 15 min. The membranes were then rinsed with PBS and running water. Finally, stained cells were photographed and counted under a light microscope to quantify migratory capacity.

### Statistical analysis

All bioinformatics analyses were conducted using the R software environment (version 4.3.2), and experimental data analysis and visualization were performed with GraphPad Prism (version 9.0). Statistical significance was defined as *P* < 0.05, unless otherwise specified.

## Results

### Differential expression of IMRGs

**Fig. 1:**
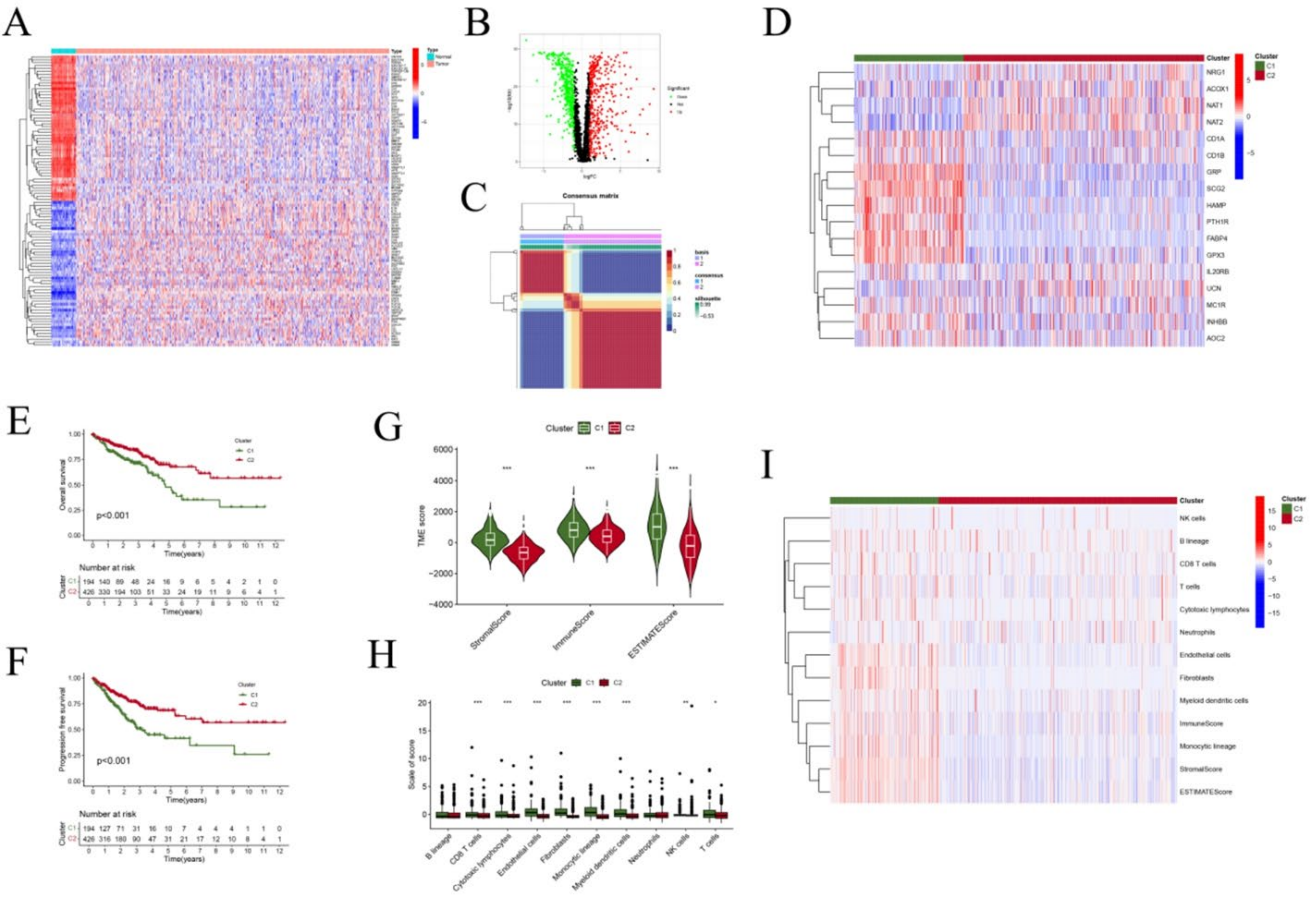
Identification of CRC molecular subtypes using the NMF algorithm. **A**, **B** Heatmaps and volcano plots showing the top 50 most significantly upregulated and downregulated IMRGs in CRC tumor samples. **C** Consensus matrix heatmap of the NMF clustering results (k = 2). **D** Heatmap illustrating IMRG expression profiles across the two molecular clusters identified by NMF. **E**, **F** K–M survival analyses comparing OS (**E**) and PFS (**F**) between the two CRC subtypes. **G** Comparison of TME scores—including StromalScore, ImmuneScore, and ESTIMATEScore—between the two clusters. Cluster 2 showed the lowest scores, with significant differences (*P* < 0.001). **H** Comparison of immune cell infiltration levels between subtypes using the MCPcounter algorithm. The bar chart depicts the relative abundance of immune cells in the TME across the two clusters. **I** Heatmap summarizing immune cell scores derived from the ESTIMATE and MCPcounter algorithms

In total, 2,652 IMRGs were identified in this study. We subsequently analyzed their expression patterns in normal and tumor CRC samples. A total of 762 genes were found to be significantly differentially expressed (FDR < 0.05, |log₂FC| > 1). To visualize these differences, heatmaps (Fig. 1A) and volcano plots (Fig. 1B) were generated, illustrating the top 50 most significantly upregulated and downregulated genes in CRC tissues compared with normal samples.

### Classification of CRC subtypes according to the NMF algorithm

To identify robust molecular subtypes of CRC, NMF clustering was performed based on differentially expressed IMRGs. As shown in Supplementary Figure S2, clustering quality was assessed across ranks (k = 2–10) using multiple metrics, including the cophenetic correlation coefficient, silhouette width, dispersion, residual sum of squares (RSS), explained variance, and sparseness. Among these, k = 2 showed the best overall stability and was selected as the optimal cluster number (Fig. 1C). The consensus heatmap (Figure S3) demonstrated two well-separated clusters with high internal consistency, and PCA (Figure S4) further confirmed clear segregation between the two subtypes.

The resulting subtypes were defined as C1 and C2. Gene expression heatmaps highlighted distinct transcriptomic patterns between C1 and C2 (Fig. 1D). Kaplan–Meier analyses revealed that patients in the C1 group had significantly poorer OS and PFS compared with those in C2 (Fig. 1E, F). Given that immune and stromal admixture may influence subtype-specific survival, we further characterized their tumor microenvironment features. Specifically, C1 exhibited significantly higher StromalScore, ImmuneScore, and ESTIMATEScore than C2 (Fig. 1G), indicating higher inferred stromal and immune components in C1. Consistently, ESTIMATE-derived tumor purity was significantly lower in C1 than in C2 (Figure S5A), supporting that C1 represents a microenvironment-rich, lower-purity subtype. Immune infiltration analysis further showed markedly higher levels of CD8⁺ T cells, total T cells, and monocytes in C1 (Fig. 1H), which was corroborated by immune marker expression heatmaps (Fig. 1I). Collectively, these results characterize C1 as an immune/stroma-enriched yet poor-prognosis molecular subtype.

To relate the NMF subtypes to established CRC molecular classifications, we next evaluated their associations with CMS and MSI status. CMS analysis demonstrated a significant difference in CMS composition between C1 and C2 (*P* = 1 × 10⁻⁵), with C1 predominantly corresponding to CMS4, whereas C2 showed relative enrichment of CMS2/CMS3 (Figure S5B). In contrast, MSI status (MSI-H vs. MSI-L/MSS, corresponding to dMMR vs. pMMR) did not differ significantly between subtypes (*P* = 0.0954; Figure S5C), suggesting that the two NMF subtypes are not primarily driven by MSI status. Importantly, in a multivariable Cox regression model including tumor purity as a covariate, the NMF subtype remained significantly associated with survival, indicating that the poorer prognosis of C1 was not solely attributable to tumor purity differences (Supplementary Table S5).

### Construction of an immune–metabolism-related prognostic signature

**Fig. 2:**
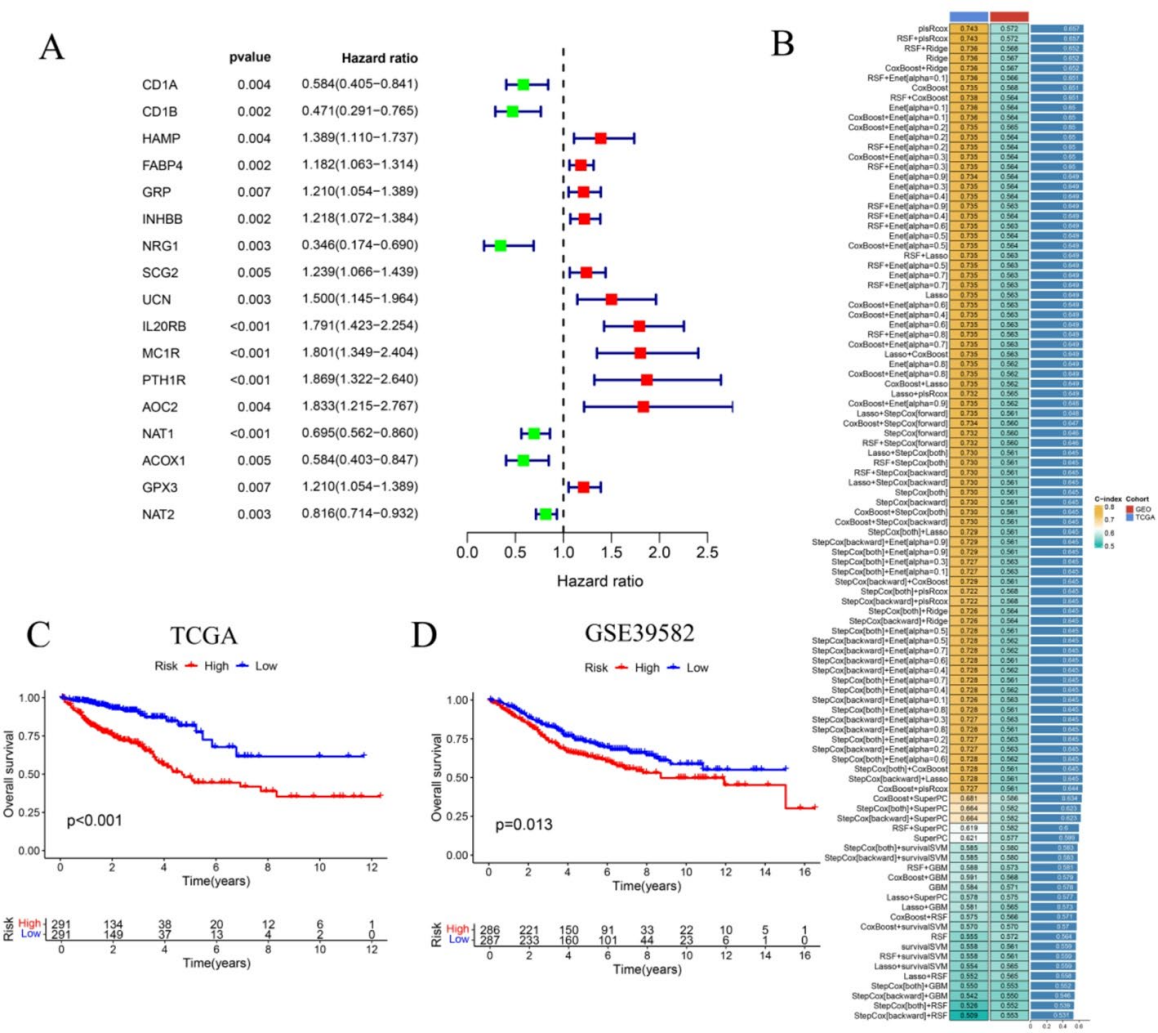
Construction of a prognostic model using machine learning. **A** Forest plot displaying IMRGs significantly associated with prognosis. **B** Identification of the optimal feature combination for model construction based on machine learning algorithms. **C**, **D** K–M survival curves illustrating the prognostic discrimination between high- and low-risk groups in the TCGA training cohort (**C**) and the GEO validation cohort (**D**)

As shown in Fig. 2A and 17 IMRGs significantly associated with prognosis were identified through univariate Cox regression analysis (*P* < 0.05). To construct a robust prognostic model, a machine learning–based ensemble framework was employed using these 17 genes as initial input features. A total of 101 predictive models were generated by combining diverse feature selection and survival modeling algorithms. Model performance was assessed using the C-index across both the TCGA training cohort and the external GEO validation dataset. As illustrated in Fig. 2B, the RSF + PLS Cox regression (plsRcox) model achieved the highest average C-index (0.657) among all evaluated methods, demonstrating superior prognostic accuracy and robustness. Ultimately, IL20RB, MC1R, PTH1R, and NAT1 were identified as the final prognostic gene signature constituting the optimized IMRG-based risk model.

### Validation of the prognostic characteristics of IMRGs

**Fig. 3:**
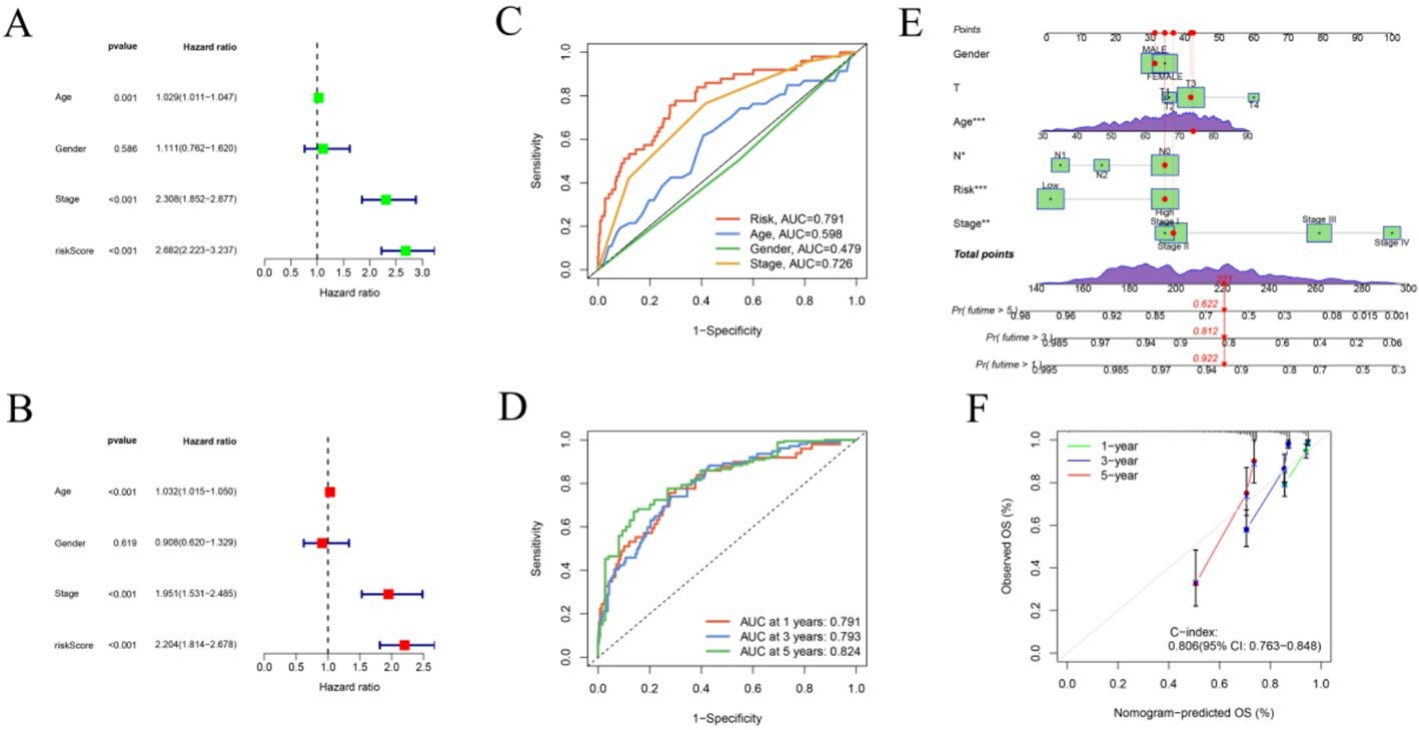
RS–based prognostic analysis and evaluation of clinical independence. **A** Univariate Cox regression analysis of prognostic factors. **B** Multivariate Cox regression analysis confirming independent prognostic predictors. **C** ROC curve evaluating the predictive performance of the 17-IMRG prognostic model. **D** Time-dependent ROC curves illustrating model performance for 1-, 3-, and 5-year OS. **E** Nomogram integrating RS, age, stage, and T and N classifications to predict 1-, 3-, and 5-year OS probabilities in CRC patients. **F** Calibration curves demonstrating concordance between predicted and observed OS at 1, 3, and 5 years

Patients were classified into high-risk and low-risk groups using the cohort-specific median RS in both the internal TCGA cohort and the external GEO validation dataset. Survival analyses performed in both the internal TCGA cohort and the external GEO validation dataset revealed a significant survival disparity between the two groups, with the high-risk group exhibiting markedly poorer outcomes (Fig. 2C, D). Forest plots (Fig. 3A–B) further demonstrated that age and cancer stage, but not gender, were independent prognostic variables significantly associated with CRC outcomes. ROC curve analysis (Fig. 3C, D) confirmed the strong predictive performance of the IMRG-based model, yielding an AUC of 0.791, surpassing that of traditional clinical prognostic indicators. Moreover, the model achieved AUC values of 0.791, 0.793, and 0.824 for 1-, 3-, and 5-year OS predictions, respectively, demonstrating its robust and consistent prognostic capability.

### Comparison of RS across clinical features

To integrate molecular and clinical information, a nomogram was constructed by combining the RS with key clinical and pathological variables to predict 1-, 3-, and 5-year OS probabilities in CRC patients. As illustrated in Fig. 3E, an example case demonstrates individualized risk estimation using this model. The calibration curve (Fig. 3F) showed excellent concordance between the predicted and observed OS rates at all evaluated time points, confirming the model’s predictive reliability.

**Fig. 4:**
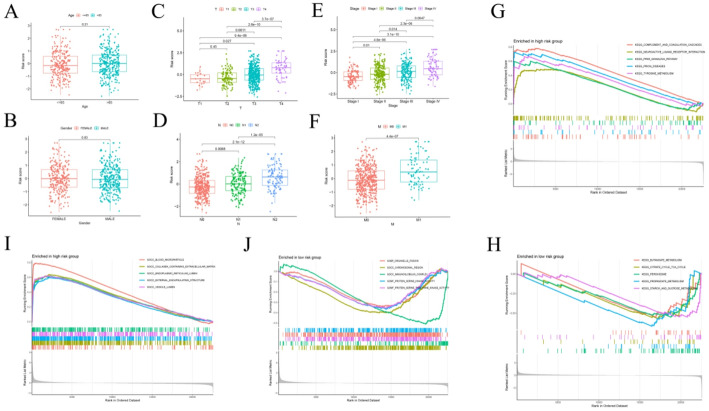
Clinical correlation and GSEA. **A**–**F** Associations between RS and clinical characteristics, including age, sex, TNM stage, and overall tumor stage. **G**–**J** GSEA results highlighting significantly enriched pathways in the high-risk and low-risk groups

To further examine the clinical relevance of the IMRG-based RS, we analyzed its association with various clinical features in the TCGA cohort using an independent sample *t*-test. RS were stratified and compared across multiple clinical subgroups. No significant differences in RS were observed with respect to patient age or gender (Fig. 4A, B). However, a progressive and significant increase in RS was noted with advancing TNM stage (Fig. 4C–F), indicating a strong relationship between tumor progression and higher risk classification. Collectively, these findings demonstrate that the IMRG-based prognostic model maintains robust predictive performance across diverse clinical conditions.

### GSEA across different risk groups in CRC

GSEA was performed to identify biological processes and signaling pathways significantly associated with the high-risk and low-risk CRC groups. In the high-risk group (Fig. 4G, I), enrichment was observed in pathways related to the complement and coagulation cascades and neuroactive ligand–receptor interactions, suggesting that these processes may play critical roles in promoting disease progression and modulating systemic inflammatory responses. Additionally, significant enrichment of genes associated with the collagen-containing extracellular matrix (ECM) was detected, indicating that ECM remodeling may contribute to increased tumor invasiveness and metastatic potential. In contrast, the low-risk group (Fig. 4H, J) displayed upregulation of pathways associated with energy metabolism, including butanoate metabolism and the citric acid (TCA) cycle, reflecting preserved metabolic homeostasis and normal cellular function. Furthermore, the enrichment of protein serine/threonine kinase activity suggested enhanced regulation of signal transduction and cell cycle control, which may contribute to greater cellular stability and slower disease progression in this subgroup.

### Immune infiltration of IMRG-based prognostic features and prediction of immunotherapy response

**Fig. 5:**
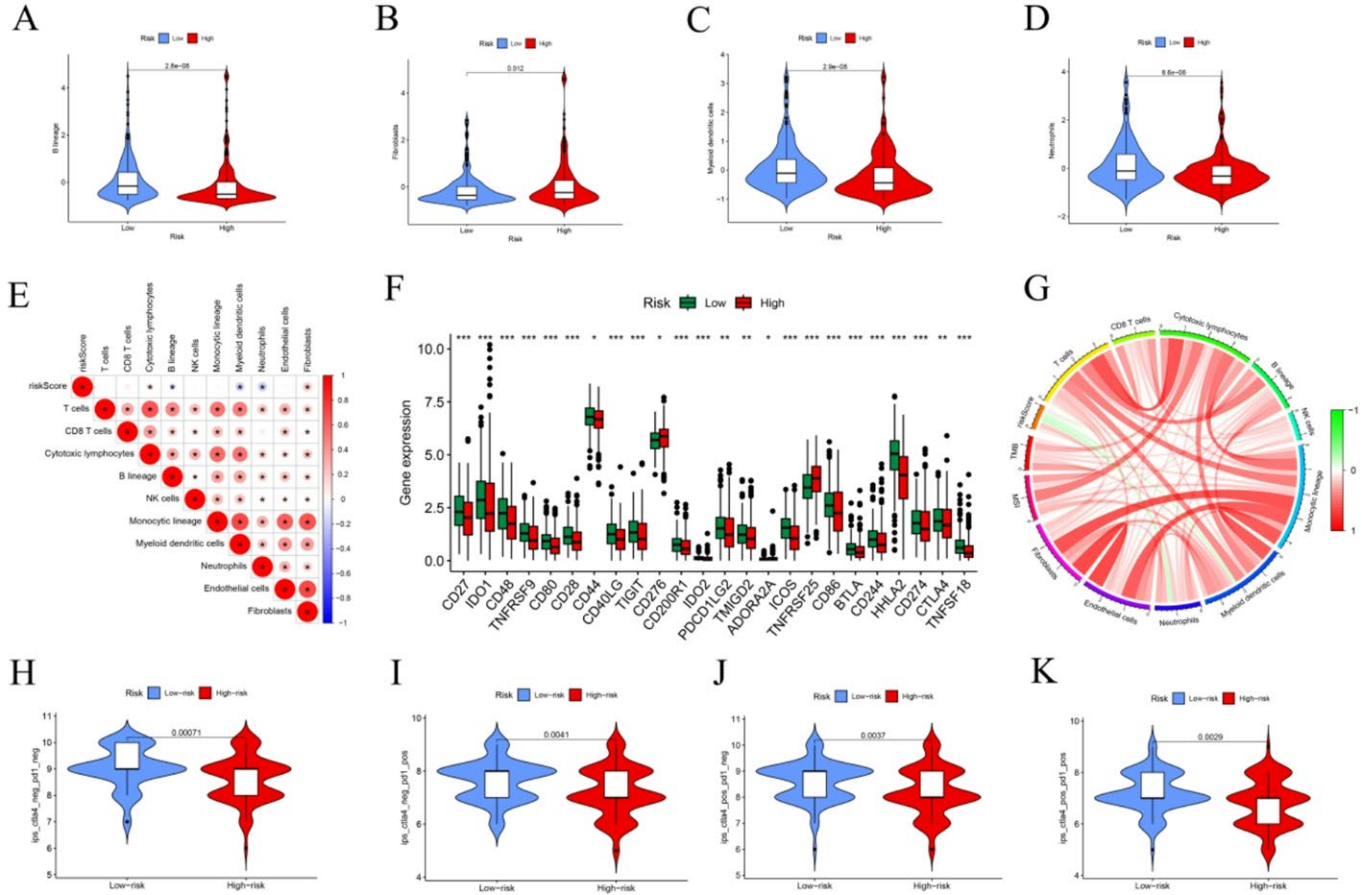
Prognostic potential of the IMRG signature and prediction of immunotherapy response. **A**–**D** Comparison of immune cell infiltration between high- and low-risk groups. **E** Heatmap depicting correlations between RS and immune cell infiltration. **F** Differential expression of immune checkpoint molecules between high- and low-risk groups. **G** Correlation analysis of RS with MSI status and immune cell populations. **H**–**K** Correlations between RS and four IPS parameters related to anti-CTLA-4, anti-PD-1, or combination ICI responses

To explore the relationship between the RS and immune cell infiltration within the TME, we assessed the abundance of multiple immune cell types across risk groups. The analysis revealed a positive correlation between RS and the infiltration levels of T cells, CD8⁺ T cells, cytotoxic lymphocytes, and fibroblasts, whereas myeloid dendritic cells and monocytes exhibited negative correlations with RS (Fig. 5A–E). These patterns were further confirmed through a chord diagram (Fig. 5G), illustrating the complex relationships between immune cell populations and the risk model. Moreover, the expression levels of most immune checkpoint molecules—with the exception of ADORA2A, CD276, and TNFRSF25—were significantly elevated in the low-risk group (Fig. 5F), suggesting enhanced immune activation in these patients. To evaluate the potential of the IMRG-based model in predicting immunotherapy outcomes, we analyzed the Immunotherapy Prediction Score (IPS) in relation to RS. Significant differences in IPS and IPS-CTLA4 values were observed between the high- and low-risk groups (Fig. 5H–K). Collectively, these results indicate that the IMRG-based prognostic model not only reflects distinct immune infiltration landscapes within the TME but also possesses strong predictive potential for patient responsiveness to immunotherapy.

### Comparison of the IMRG-based prognostic model with other published models

**Fig. 6:**
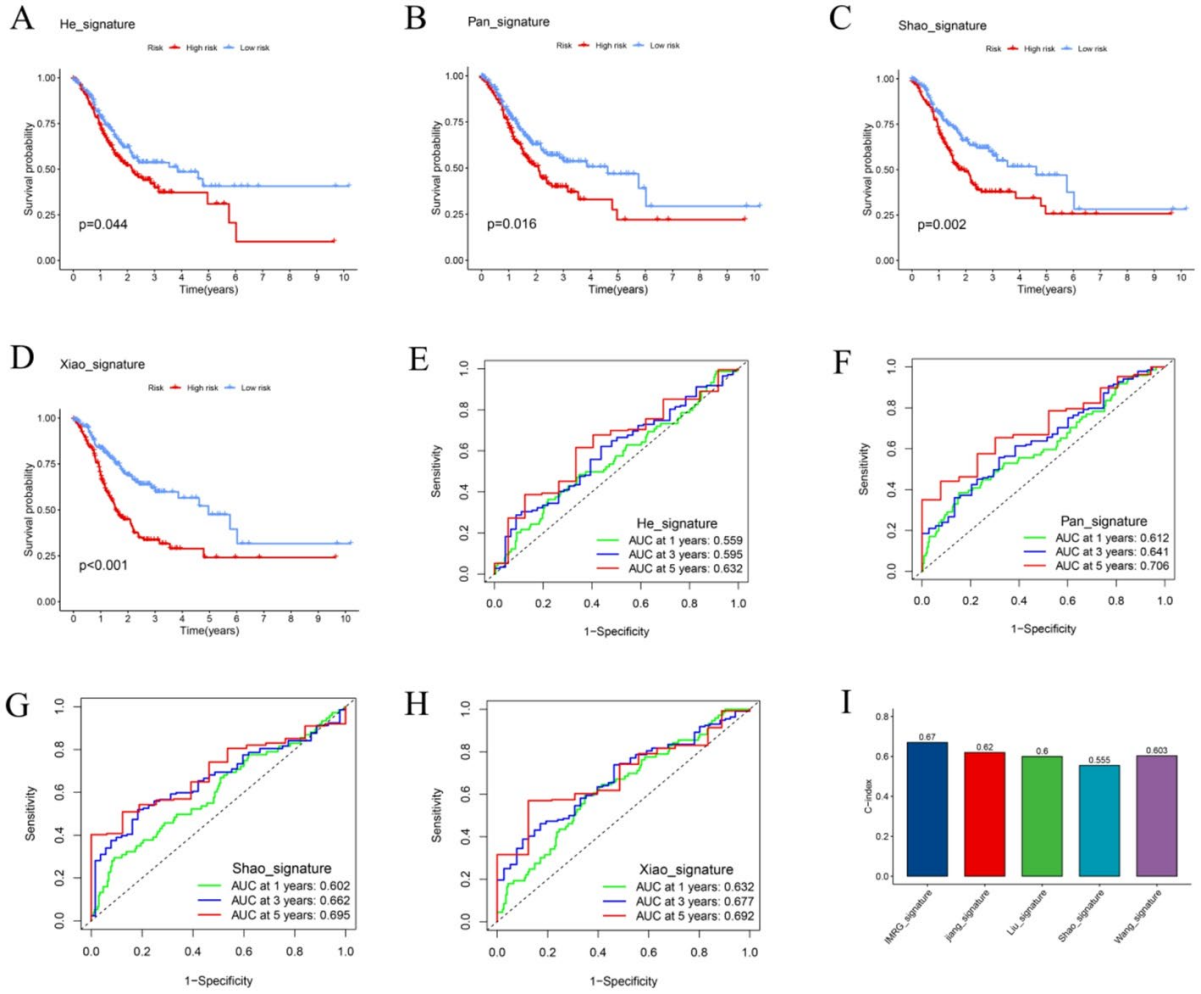
Comparative evaluation of the IMRG prognostic model and previously published signatures. **A**–**D** K–M survival analyses of four previously published prognostic signatures. **E**–**H** Time-dependent ROC curves comparing the predictive accuracy of the four signatures. **I** Comparison of the concordance index (C-index) values among the IMRG model and the four reference models, showing superior performance of the IMRG signature

To evaluate the predictive performance of our newly developed IMRG-based prognostic model, we compared it against four previously published prognostic models using the TCGA cohort as a reference dataset [19–22]. A standardized method was applied to compute and normalize the RS across all models, ensuring consistent comparability. As shown in Fig. 6A–D, each of the four existing models effectively stratified CRC patients into high-risk and low-risk groups, with statistically significant differences in survival between the two categories. However, ROC curve analyses demonstrated that the IMRG-based model exhibited superior predictive accuracy, achieving AUC values of 0.791, 0.793, and 0.824 for 1-, 3-, and 5-year OS, respectively (Fig. 6E–H). Furthermore, the C-index of the IMRG model reached 0.67, exceeding those of the other four models (0.62, 0.60, 0.555, and 0.603, respectively; Fig. 6I). Collectively, these results highlight the superior prognostic performance and robust discriminative capability of the IMRG-based model in predicting long-term survival outcomes compared with previously established models.

### Differences in drug sensitivity between high- and low-risk groups

To explore potential therapeutic implications of the IMRG-based risk model, we compared the chemotherapeutic drug sensitivity between patients in the high-risk and low-risk groups. The analysis revealed that patients classified as high-risk exhibited lower IC₅₀ values for most chemotherapy agents, indicating greater drug sensitivity (Figure S6).

### IL20RB is significantly upregulated in CRC tissues and cell lines

**Fig. 7:**
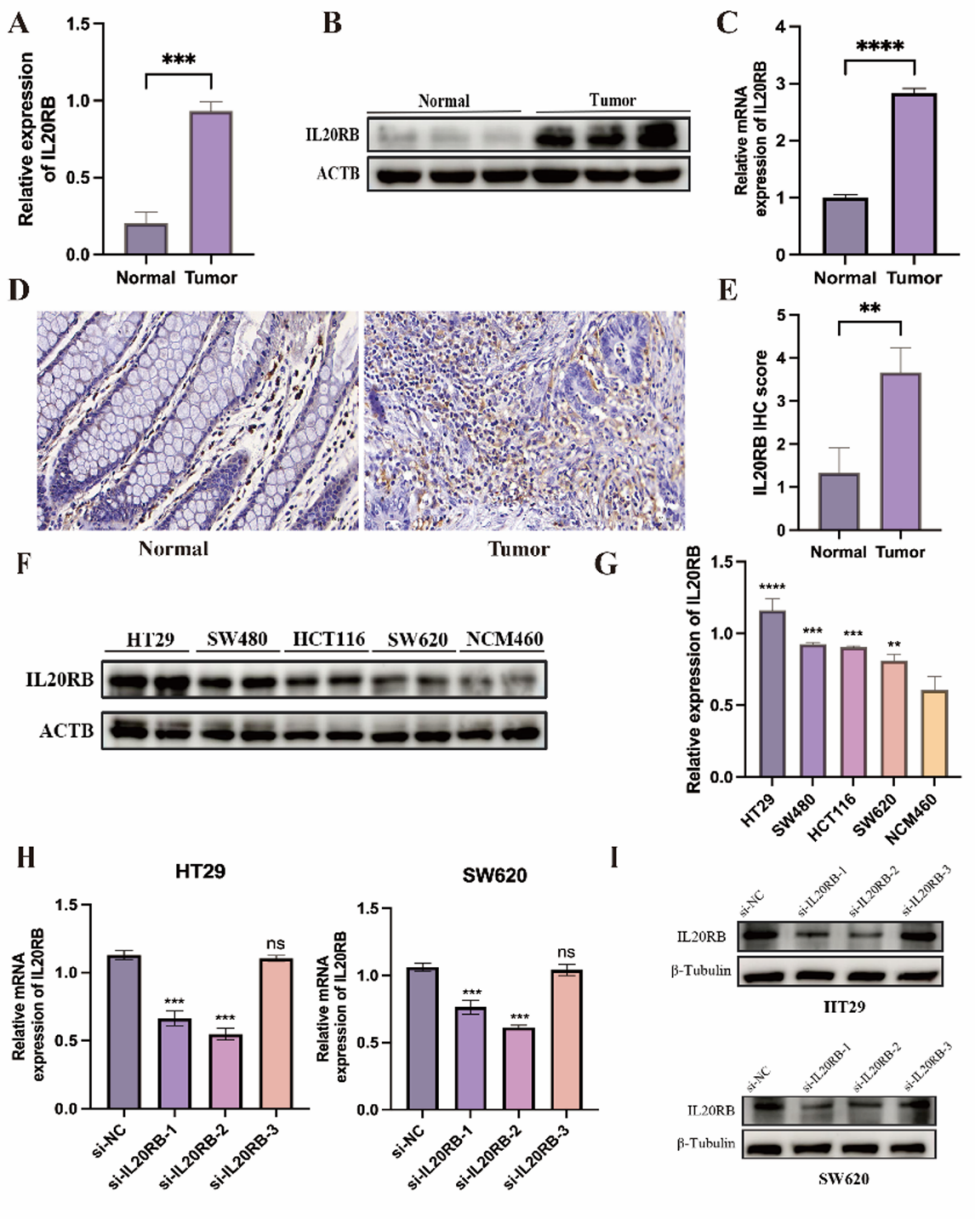
Expression and validation of IL20RB in CRC tissues and cell lines. **A** qRT–PCR analysis showing significantly upregulated IL20RB mRNA expression in CRC tissues compared with adjacent normal tissues. **B**, **C** Western blot analysis and corresponding densitometric quantification confirming increased IL20RB protein expression in CRC tissues. **D**, **E** Immunohistochemistry (IHC) demonstrating strong cytoplasmic and membranous IL20RB staining in CRC epithelial cells, while normal intestinal glands exhibited weak or negligible staining. **F**–**G** Western blot and quantification revealing elevated IL20RB expression in CRC cell lines (HT29, SW480, HCT116, and SW620) compared with the normal colonic epithelial cell line (NCM460). **H**–**I** Validation of IL20RB knockdown efficiency in HT29 and SW620 cells by qRT–PCR and Western blot, showing significant suppression of IL20RB expression by si-IL20RB-1 and si-IL20RB-2, whereas si-IL20RB-3 showed minimal effect. Data are presented as mean ± SD. Statistical significance: *P < 0.05, **P < 0.01, ***P < 0.001, ****P < 0.0001

qRT–PCR analysis revealed that IL20RB mRNA expression was significantly higher in CRC tissues compared with normal intestinal epithelium (*P* < 0.001; Fig. 7A). Consistently, WB confirmed increased IL20RB protein levels in tumor tissues (Fig. 7B), further supported by densitometric quantification (*P* < 0.01; Fig. 7C). IHC demonstrated strong cytoplasmic and membranous IL20RB staining in CRC epithelial cells, whereas normal intestinal glands exhibited weak or negligible expression (Fig. 7D, E). Across CRC cell lines, IL20RB expression was markedly elevated in HT29, SW480, HCT116, and SW620 cells relative to the normal epithelial cell line NCM460 (Figs. 7F–G). To assess gene silencing efficiency, three small interfering RNAs (siRNAs) targeting IL20RB were transfected into HT29 and SW620 cells. Among these, si-IL20RB-1 and si-IL20RB-2 significantly reduced IL20RB expression at both the mRNA and protein levels, whereas si-IL20RB-3 exhibited minimal inhibitory effects (Fig. 7H–I). Consequently, si-IL20RB-1 and si-IL20RB-2 were selected for subsequent functional assays in these cell lines.

### Silencing of IL20RB inhibits proliferation, migration, and invasion of CRC cells

**Fig. 8:**
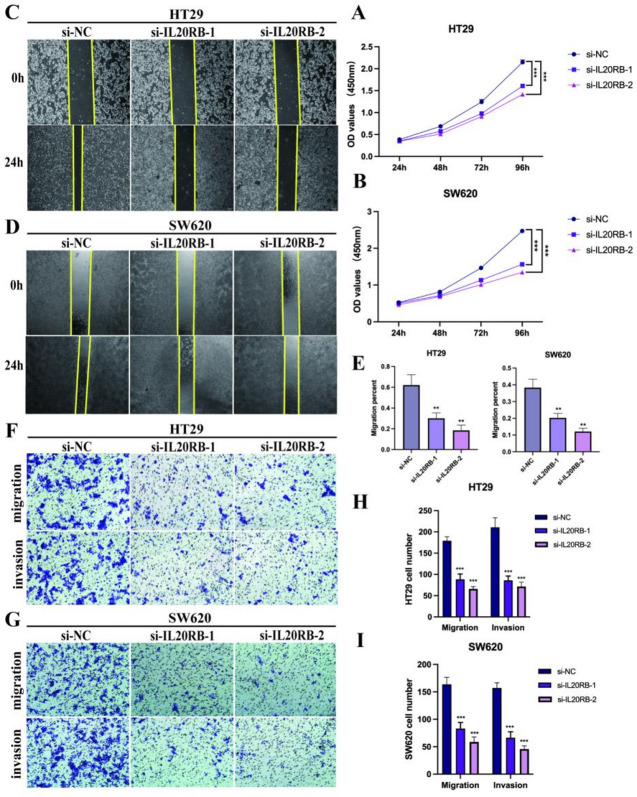
Silencing of IL20RB suppresses proliferation, migration, and invasion in CRC cells. **A**, **B** CCK-8 assays showing that IL20RB knockdown significantly reduced the proliferation of HT29 and SW620 cells at 72 h and 96 h. **C**, **D** Representative wound-healing images demonstrating markedly decreased migration capacity following IL20RB silencing in HT29 and SW620 cells at 24 h. **E** Quantitative analysis of wound closure percentage showing that IL20RB knockdown significantly inhibited cell migration (*P* < 0.01). **F**–**G** Transwell migration and invasion assays illustrating fewer migrated and invaded cells in IL20RB-silenced HT29 and SW620 cells compared with controls. **H**, **I** Quantification of migrated and invaded cells confirming that IL20RB depletion significantly reduced both migration and invasion in HT29 and SW620 cells. Data are presented as mean ± SD. Statistical significance: *P < 0.05, **P < 0.01, ***P < 0.001, ****P < 0.0001

CCK-8 assays demonstrated that IL20RB knockdown significantly suppressed the proliferation of HT29 and SW620 CRC cells compared with their respective control groups (*P* < 0.001; Fig. 8A, B). Wound-healing assays revealed that cell motility was markedly reduced in both cell lines following IL20RB silencing at 24 h (Fig. 8C–E). Furthermore, Transwell migration and invasion assays showed that the numbers of migrating and invading cells were significantly decreased after IL20RB depletion (*P* < 0.01; Fig. 8F–I). Collectively, these results indicate that IL20B promotes CRC cell proliferation, migration, and invasion, and that its silencing markedly impairs these malignant phenotypes.

## Discussion

Cancer cells undergo extensive metabolic reprogramming, a hallmark of tumorigenesis, which enables them to sustain growth and survival under adverse conditions [23]. In CRC, this metabolic adaptation facilitates energy acquisition and the biosynthesis of essential macromolecules required for rapid proliferation [24]. These alterations include enhanced glycolysis, reprogrammed lipid metabolism, and increased amino acid utilization, collectively supporting tumor progression under conditions of hypoxia and nutrient deprivation [25, 26]. Beyond energy metabolism, these changes contribute to immune evasion and confer resistance to chemotherapy and radiotherapy [27]. With the advent of high-throughput sequencing technologies, integrative genomic analyses have provided deeper insights into the molecular heterogeneity of CRC, refining both therapeutic strategies and prognostic evaluation [28]. Previous studies have shown that MRGs and IRGs play pivotal roles in CRC pathogenesis, leading to the development of prognostic models and therapeutic markers based on their combined expression patterns [29]. Despite sharing common clinical features, CRC patients display marked molecular heterogeneity, accounting for substantial differences in treatment responses and survival outcomes. Integrating metabolic and immune signatures into a unified prognostic framework has proven to enhance predictive accuracy and clinical applicability [30]. Building on these advances, our study employed machine learning–based approaches to identify and validate IMRGs as prognostic biomarkers, aiming to improve risk stratification and delineate the immunological landscape of CRC.

In this study, we analyzed the transcriptional expression profiles of IMRGs to classify CRC patients into distinct molecular subtypes. These subtypes exhibited significant differences in both survival outcomes and immune landscape characteristics. Notably, patients belonging to Cluster 1 demonstrated poorer survival rates compared with those in Cluster 2. Cluster 1 was further characterized by enhanced immune cell infiltration and a more complex TME, which may collectively contribute to the unfavorable clinical prognosis observed in this group. Although immune infiltration is often correlated with improved patient outcomes, our findings suggest that in certain CRC subtypes, a highly infiltrated TME may paradoxically be associated with worse survival. This may result from the dominance of immunosuppressive cellular components within the TME. In Cluster 1, we observed elevated levels of myeloid dendritic cells, fibroblasts, and endothelial cells, which are known to facilitate immune tolerance, angiogenesis, and stromal remodeling—processes that can enhance tumor progression [31]. Specifically, the accumulation of myeloid dendritic cells may promote an immunosuppressive milieu, while fibroblasts and endothelial cells contribute to tumor growth by providing structural and metabolic support. Given the clear potential of IMRGs in CRC classification, we applied univariate Cox regression and a machine learning–based ensemble framework to identify 17 IMRGs significantly associated with patient prognosis. Using these features, we developed a prognostic risk model capable of stratifying CRC patients into distinct risk categories and predicting OS with high accuracy. Importantly, this model also demonstrated potential utility in assessing immunotherapy responsiveness and was further validated in an independent external dataset, confirming its robustness and generalizability.

Within the TME, the infiltration and activity of immune cells play pivotal roles in tumor initiation, progression, and therapeutic response. These immune populations not only participate in immune surveillance but also modulate immune evasion mechanisms through complex interactions with tumor and stromal cells [32]. Among them, CD8⁺ T cells serve as the primary cytotoxic effector cells, recognizing and eliminating tumor cells that express specific antigens. This process is initiated when the T cell receptor (TCR) binds to antigenic peptides presented by major histocompatibility complex class I (MHC-I) molecules on tumor cell surfaces, enabling CD8⁺ T cells to accurately target and destroy malignant cells [33]. However, immunosuppressive factors within the TME—such as immune checkpoint molecules and tumor-associated fibroblasts—can attenuate CD8⁺ T-cell activity, facilitating tumor immune escape [34]. In our study, despite higher infiltration of T cells and CD8⁺ T cells in the high-risk group, the immune checkpoint landscape exhibited a heterogeneous pattern. Most checkpoint molecules were more highly expressed in the low-risk group, consistent with an immune-inflamed phenotype, whereas ADORA2A and CD276 were relatively elevated in the high-risk group. Prior evidence suggests that ADORA2A- and CD276-associated signaling may impair T-cell activation, potentially through cAMP/PKA-related pathways and downstream inhibition of transcriptional programs such as NF-κB and AP-1 [35, 36]. Accordingly, we propose that the unfavorable prognosis in the high-risk subgroup may relate to selective activation of specific inhibitory axes (e.g., ADORA2A/CD276) rather than a global increase in checkpoint activity. Nevertheless, because T-cell exhaustion states and pathway activity were not directly interrogated here, this interpretation should be regarded as hypothesis-generating and warrants further mechanistic validation.

Our study identified four IMRGs—IL20RB, MC1R, PTH1R, and NAT1—that were closely associated with CRC prognosis in our predictive model. Among them, IL20RB functions as a receptor for the cytokines IL-20 and IL-22, forming a functional heterodimeric receptor complex through binding with IL20RA or IL22RA1. This receptor complex mediates signaling cascades initiated by multiple interleukins, including IL-19, IL-20, and IL-24 [37]. Previous studies in breast cancer have demonstrated that IL20RB regulates the distribution of inflammatory mediators and immune cells within the TME [38]. Overexpression of IL-20 in breast cancer promotes the recruitment and activation of pro-inflammatory macrophages through IL20RB, which in turn secrete cytokines and chemokines that facilitate tumor growth and metastasis [39]. Moreover, IL20RB-mediated signaling can directly enhance the survival and proliferation of tumor cells by activating pathways such as NF-κB and STAT3, thereby increasing their anti-apoptotic capacity [40]. MC1R encodes a G protein-coupled receptor that regulates melanin biosynthesis, and its dysfunction has been strongly linked to skin cancer susceptibility. In melanoma, MC1R modulates the cellular response to ultraviolet (UV) radiation through control of melanogenic pathways and may also participate in immune surveillance by influencing antigen presentation or immune signaling [41]. PTH1R, the receptor for parathyroid hormone (PTH) and PTH-related protein (PTHrP), plays a central role in calcium–phosphorus homeostasis and bone metabolism. In oncology, PTH1R is implicated in bone metastasis in cancers such as breast and prostate cancer, where it contributes to the remodeling of the bone microenvironment [42]. NAT1, an enzyme involved in xenobiotic biotransformation, exerts multifaceted effects on tumor metabolism and immune regulation. Elevated NAT1 expression has been associated with enhanced glycolysis and altered drug metabolism, potentially promoting chemoresistance in CRC [43]. Furthermore, NAT1-derived metabolites may modify the TME, affecting immune cell function and facilitating tumor immune evasion [44]. Collectively, these findings underscore the importance of further elucidating the mechanistic roles of IL20RB, MC1R, PTH1R, and NAT1 in CRC, particularly their interplay between metabolic regulation and immune modulation. Such insights may provide a foundation for developing novel targeted therapies and improving the efficacy of existing treatment regimens in CRC.

Despite the strengths of this study, several limitations should be acknowledged. First, the prognostic indicators identified were derived solely from publicly available datasets, such as TCGA and GEO. Although these findings demonstrate substantial predictive power, a deeper understanding of the biological roles of IMRGs in CRC progression requires further in vitro and in vivo validation. Moreover, the influence of these IMRG-based prognostic signatures on the efficacy of targeted therapy, chemotherapy, and immunotherapy remains to be comprehensively assessed in prospective clinical studies. Finally, future research involving larger, multi-center, and ethnically diverse cohorts will be essential to verify the robustness and generalizability of the proposed prognostic model across different clinical settings. Mechanistically, we did not conduct rescue experiments or directly assess downstream signaling activity; therefore, the proposed pathway-level interpretation should be considered hypothesis-generating and warrants further validation.

## Supplementary Information

Supplementary Material 1.

Supplementary Material 2.

Supplementary Material 3.

Supplementary Material 4.

Supplementary Material 5.

Supplementary Material 6.

Supplementary Material 7.

## Data Availability

The datasets presented in this study can be found in online repositories. The names of the repository/repositories and accession number(s) can be found below: https://portal.gdc.cancer.gov, The Cancer Genome Atlas; https://www.ncbi.nlm.nih.gov/geo, The Gene Expression Omnibus (GSE39582).

## References

[1] Dedieu S, Bouché O. Clinical, pathological, and molecular characteristics in colorectal cancer. Cancers (Basel). 2022;14(23):5958. 36497440 10.3390/cancers14235958 PMC9739916

[2] Artemaki PI, Kontos CK. Editorial for the special issue molecular biomarkers in colorectal adenocarcinoma. Int J Mol Sci. 2021;22(4):2052. 33669582 10.3390/ijms22042052 PMC7922430

[3] Joonsalu M, Mägi M, Kase M, Jõgi T, Tammaru M, Ojamaa K, Asser T, Jaal J. Peaaju primaarsetesse pahaloomulistesse kasvajatesse haigestumus 15–44aastaste Eesti noorte hulgas ajavahemikul 1980–2009. 2014.

[4] Paul B, O’Neil BH, McRee AJ. Checkpoint inhibition for colorectal cancer: progress and possibilities. Immunotherapy. 2016;8(6):693–704. 27197538 10.2217/imt-2016-0013

[5] Morgan E, Arnold M, Gini A, Lorenzoni V, Cabasag CJ, Laversanne M, Vignat J, Ferlay J, Murphy N, Bray F. Global burden of colorectal cancer in 2020 and 2040: incidence and mortality estimates from GLOBOCAN. Gut. 2023;72(2):338–44. 36604116 10.1136/gutjnl-2022-327736

[6] Feng Y, Jin H, Guo K, Wasan HS, Ruan S, Chen C. Causes of death after colorectal cancer diagnosis: a population-based study. Front Oncol. 2021;11:647179. 33859947 10.3389/fonc.2021.647179 PMC8042257

[7] Matteucci L, Bittoni A, Gallo G, Ridolfi L, Passardi A. Immunocheckpoint inhibitors in microsatellite-stable or proficient mismatch repair metastatic colorectal cancer: are we entering a new era? Cancers (Basel). 2023;15(21): 5189. 37958363 10.3390/cancers15215189 PMC10648369

[8] Ding K, Mou P, Wang Z, Liu S, Liu J, Lu H, et al. The next bastion to be conquered in immunotherapy: microsatellite stable colorectal cancer. Front Immunol. 2023;14:1298524. 38187388 10.3389/fimmu.2023.1298524 PMC10770832

[9] Zhang H, Zhang G, Xu P, Yu F, Li L, Huang R, et al. Optimized dynamic network biomarker deciphers a high-resolution heterogeneity within thyroid cancer molecular subtypes. Med Res. 2025;1(1):10–31.

[10] Pavlova NN, Zhu J, Thompson CB. The hallmarks of cancer metabolism: still emerging. Cell Metab. 2022;34(3):355–77. 35123658 10.1016/j.cmet.2022.01.007 PMC8891094

[11] Fukushi A, Kim H-D, Chang Y-C, Kim C-H. Revisited metabolic control and reprogramming cancers by means of the Warburg effect in tumor cells. Int J Mol Sci. 2022;23(17):10037. 36077431 10.3390/ijms231710037 PMC9456516

[12] Frisardi V, Canovi S, Vaccaro S, Frazzi R. The significance of microenvironmental and circulating lactate in breast cancer. Int J Mol Sci. 2023;24(20):15369. 37895048 10.3390/ijms242015369 PMC10607673

[13] Ke X, Li K, Jiang A, Zhang Y, Wang Q, Li Z, et al. Cloud-based GWAS platform: an innovative solution for efficient acquisition and analysis of genomic data. Med Res. 2025;1(3):397–411.

[14] Zhang Y, Zhai Z, Duan J, Wang X, Zhong J, Wu L, Li A, Cao M, Wu Y, Shi H, et al. Lactate: the mediator of metabolism and immunosuppression. Front Endocrinol (Lausanne). 2022;13: 901495. 35757394 10.3389/fendo.2022.901495 PMC9218951

[15] Chen J, Wang R, Liu Z, Fan J, Liu S, Tan S, et al. Unbalanced glutamine partitioning between CD8T cells and cancer cells accompanied by immune cell dysfunction in hepatocellular carcinoma. Cells. 2022;11(23):3924. 36497182 10.3390/cells11233924 PMC9739589

[16] Hosseinalizadeh H, Mahmoodpour M, Samadani AA, Roudkenar MH. The immunosuppressive role of indoleamine 2, 3-dioxygenase in glioblas toma: mechanism of action and immunotherapeutic strategies. Med Oncol. 2022;39(9):130. 35716323 10.1007/s12032-022-01724-w PMC9206138

[17] Ganjoo S, Gupta P, Corbali HI, Nanez S, Riad TS, Duong LK, et al. The role of tumor metabolism in modulating T-cell activity and in optimizing immunotherapy. Front Immunol. 2023;14:1172931. 37180129 10.3389/fimmu.2023.1172931 PMC10169689

[18] Zhang P, Zhang M, Liu J, Zhou Z, Zhang L, Luo P, Zhang Z. Mitochondrial pathway signature (MitoPS) predicts immunotherapy response and reveals NDUFB10 as a key immune regulator in lung adenocarcinoma. J Immunother Cancer. 2025;13(7): e012069. 40744665 10.1136/jitc-2025-012069 PMC12315039

[19] Xiao Y, Zhang G, Wang L, Liang M. Exploration and validation of a combined immune and metabolism gene signature for prognosis prediction of colorectal cancer. Front Endocrinol (Lausanne). 2022;13:1069528. 36518242 10.3389/fendo.2022.1069528 PMC9742469

[20] Pan B, Yue Y, Ding W, Sun L, Xu M, Wang S. A novel prognostic signatures based on metastasis- and immune-related gene pairs for colorectal cancer. Front Immunol. 2023;14:1161382. 37180113 10.3389/fimmu.2023.1161382 PMC10169605

[21] He R, Zhang H, Zhao H, Yin X, Lu J, Gu C, Gao J, Xu Q. Multiomics analysis reveals cuproptosis-related signature for evaluating prognosis and immunotherapy efficacy in colorectal cancer. Cancers (Basel). 2023;15(2):387. 36672336 10.3390/cancers15020387 PMC9856392

[22] Shao Y, Fan X, Yang X, Li S, Huang L, Zhou X, et al. Impact of cuproptosis-related markers on clinical status, tumor immune microenvironment and immunotherapy in colorectal cancer: a multi-omic analysis. Comput Struct Biotechnol J. 2023;21:3383–403. 37389187 10.1016/j.csbj.2023.06.011 PMC10300104

[23] Nong S, Han X, Xiang Y, Qian Y, Wei Y, Zhang T, et al. Metabolic reprogramming in cancer: mechanisms and therapeutics. MedComm (2020). 2023;4(2):e218. 36994237 10.1002/mco2.218 PMC10041388

[24] Zhang J, Zou S, Fang L. Metabolic reprogramming in colorectal cancer: regulatory networks and therapy. Cell Biosci. 2023;13(1):25. 36755301 10.1186/s13578-023-00977-w PMC9906896

[25] Satoh K, Yachida S, Sugimoto M, Oshima M, Nakagawa T, Akamoto S, et al. Global metabolic reprogramming of colorectal cancer occurs at adenoma stage and is induced by MYC. Proc Natl Acad Sci U S A. 2017;114(37):E7697–706. 28847964 10.1073/pnas.1710366114 PMC5604037

[26] Shen Y, Sun M, Zhu J, Wei M, Li H, Zhao P, et al. Tissue metabolic profiling reveals major metabolic alteration in color ectal cancer. Mol Omics. 2021;17(3):464–71. 33881127 10.1039/d1mo00022e

[27] Sellitto A, Pecoraro G, Giurato G, Nassa G, Rizzo F, Saggese P, et al. Regulation of metabolic reprogramming by long non-coding RNAs in cancer. Cancers (Basel). 2021;13(14):3485. 34298698 10.3390/cancers13143485 PMC8308086

[28] Ohshima K, Morii E. Metabolic reprogramming of cancer cells during tumor progression and m etastasis. Metabolites. 2021;11(1):28. 33401771 10.3390/metabo11010028 PMC7824065

[29] La Vecchia S, Sebastián C. Metabolic pathways regulating colorectal cancer initiation and progression. Semin Cell Dev Biol. 2020;98:63–70. 31129171 10.1016/j.semcdb.2019.05.018

[30] Giacomini I, Montopoli M. Editorial: metabolism meets function: the multifaced role of metabolism in cancer. Front Oncol. 2022;12:906421. 35600343 10.3389/fonc.2022.906421 PMC9115570

[31] Peng Z, Ren Z, Tong Z, Zhu Y, Zhu Y, Hu K. Interactions between MFAP5 + fibroblasts and tumor-infiltrating myeloid cells shape the malignant microenvironment of colorectal cancer. J Transl Med. 2023;21(1):405. 37344903 10.1186/s12967-023-04281-6 PMC10286363

[32] Chen DS, Mellman I. Elements of cancer immunity and the cancer-immune set point. Nature. 2017;541(7637):321–30. 28102259 10.1038/nature21349

[33] Schietinger A, Greenberg PD. Tolerance and exhaustion: defining mechanisms of T cell dysfunction. Trends Immunol. 2014;35(2):51–60. 24210163 10.1016/j.it.2013.10.001 PMC3946600

[34] Pardoll DM. The blockade of immune checkpoints in cancer immunotherapy. Nat Rev Cancer. 2012;12(4):252–64. 22437870 10.1038/nrc3239 PMC4856023

[35] Cekic C, Linden J. Adenosine A2A receptors intrinsically regulate CD8 + T cells in the tum or microenvironment. Cancer Res. 2014;74(24):7239–49. 25341542 10.1158/0008-5472.CAN-13-3581 PMC4459794

[36] Getu AA, Tigabu A, Zhou M, Lu J, Fodstad Ø, Tan M. New frontiers in immune checkpoint B7-H3 (CD276) research and drug dev elopment. Mol Cancer. 2023;22(1):43. 36859240 10.1186/s12943-023-01751-9 PMC9979440

[37] Katara GK, Kulshrestha A, Schneiderman S, Riehl V, Ibrahim S, Beaman KD. Interleukin-22 promotes development of malignant lesions in a mouse model of spontaneous breast cancer. Mol Oncol. 2020;14(1):211–24. 31725949 10.1002/1878-0261.12598 PMC6944104

[38] Hsu Y-H, Hsing C-H, Li C-F, Chan C-H, Chang M-C, Yan J-J, Chang M-S. Anti-IL-20 monoclonal antibody suppresses breast cancer progression and bone osteolysis in murine models. J Immunol. 2012;188(4):1981–91. 22238453 10.4049/jimmunol.1102843

[39] Chen Y-Y, Li C-F, Yeh C-H, Chang M-S, Hsing C-H. Interleukin-19 in breast cancer. Clin Dev Immunol. 2013;2013:294320. 23710200 10.1155/2013/294320 PMC3654677

[40] Markota A, Endres S, Kobold S. Targeting interleukin-22 for cancer therapy. Hum Vaccin Immunother. 2018;14(8):2012–5. 29617184 10.1080/21645515.2018.1461300 PMC6149728

[41] García-Borrón JC, Abdel-Malek Z, Jiménez-Cervantes C. MC1R, the cAMP pathway, and the response to solar UV: extending the horizon beyond pigmentation. Pigment Cell Melanoma Res. 2014;27(5):699–720. 24807163 10.1111/pcmr.12257 PMC4150834

[42] von Moos R, Lewis K, Massey L, Marongiu A, Rider A, Seesaghur A. Initiation of bone-targeted agents in patients with bone metastases and breast or castrate-resistant prostate cancer actively treated in rou tine clinical practice in Europe. Bone. 2022;154:116243. 34757213 10.1016/j.bone.2021.116243

[43] Carlisle SM, Trainor PJ, Doll MA, Stepp MW, Klinge CM, Hein DW. Knockout of human arylamine N-acetyltransferase 1 (NAT1) in MDA-MB-231 breast cancer cells leads to increased reserve capacity, maximum mito chondrial capacity, and glycolytic reserve capacity. Mol Carcinog. 2018;57(11):1458–66. 29964355 10.1002/mc.22869 PMC6353662

[44] Ren Y, Kumar A, Das JK, Peng H-Y, Wang L, Balllard D, Xiong X, Ren X, Zhang Y, Yang J-M, et al. Tumorous expression of NAC1 restrains antitumor immunity through the LDHA-mediated immune evasion. J Immunother Cancer. 2022;10(9):e004856. 36150745 10.1136/jitc-2022-004856 PMC9511653

