# Supplementary material for: Integrative machine learning models reveal immune and metabolic signatures predictive of colorectal cancer prognosis
Source: Discov Oncol. 2026 Mar 3;17:742. doi: 10.1007/s12672-026-04758-y (PMC13187096; doi:10.1007/s12672-026-04758-y)
Supplement: Supplementary file 1 — Supplementary Material 1. [file 12672_2026_4758_MOESM1_ESM.docx]

**Figure S1. TCGA and GEO batch effect removal.**

| **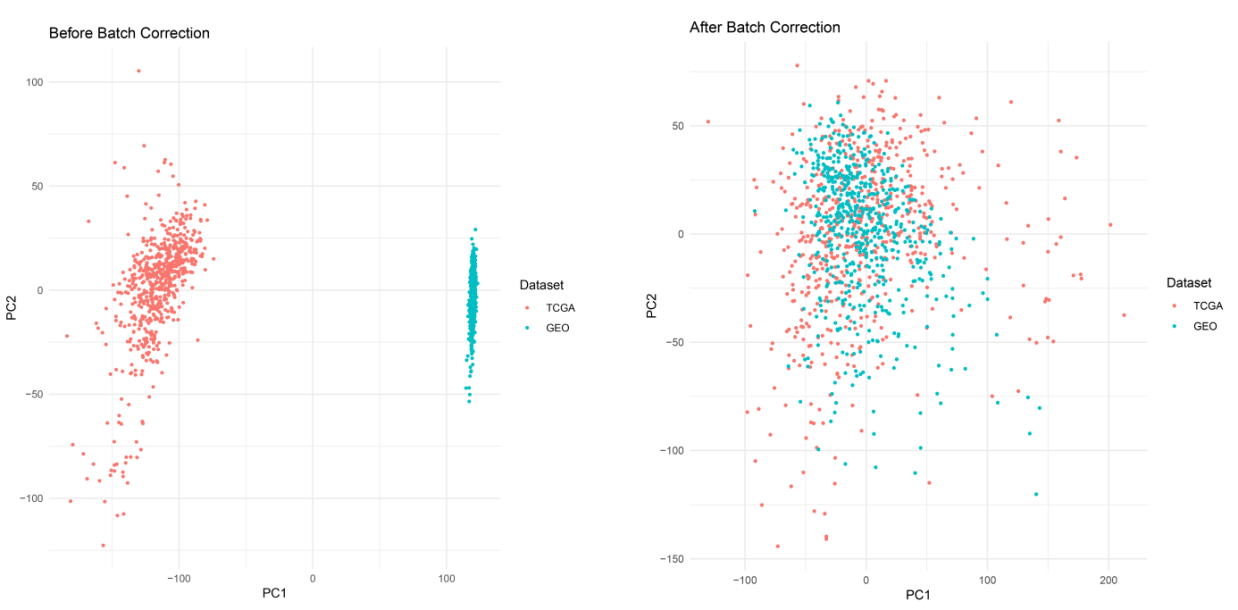**  **Figure S2. NMF rank survey for determining optimal cluster number.**  **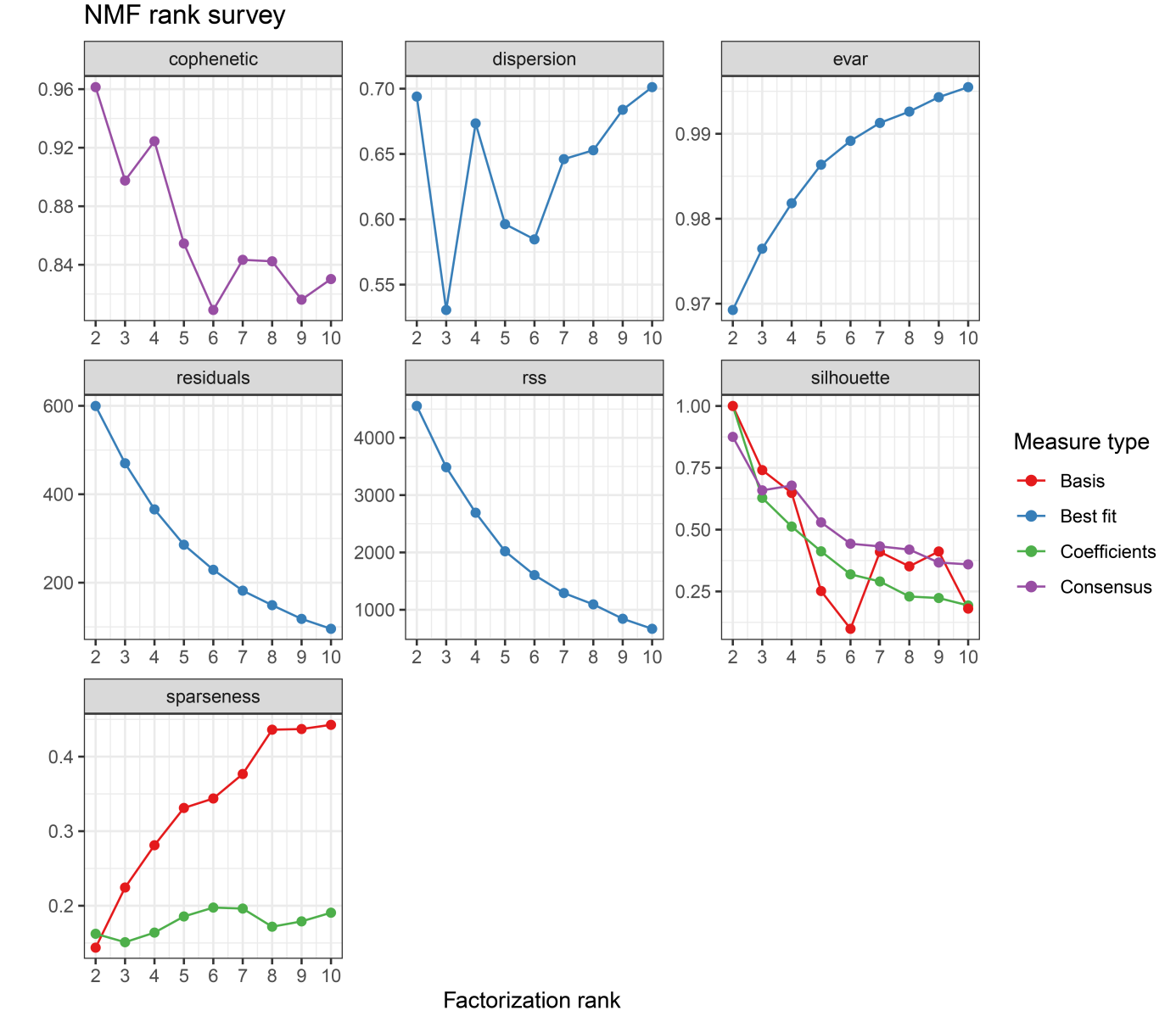**  **Figure S3. Consensus clustering heatmaps for k = 2–10.**  **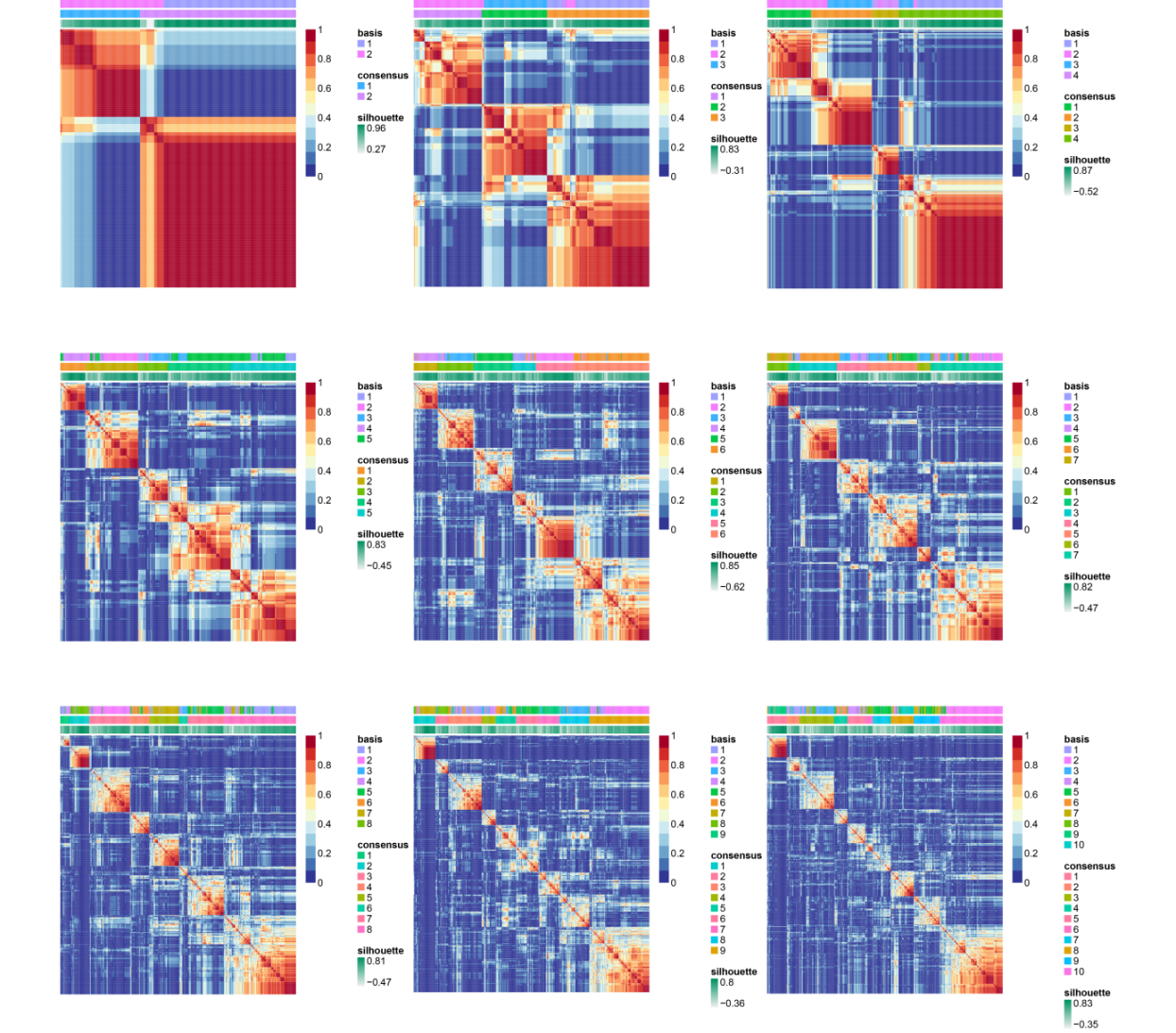**  **Figure S4. PCA of NMF-defined subtypes.**  **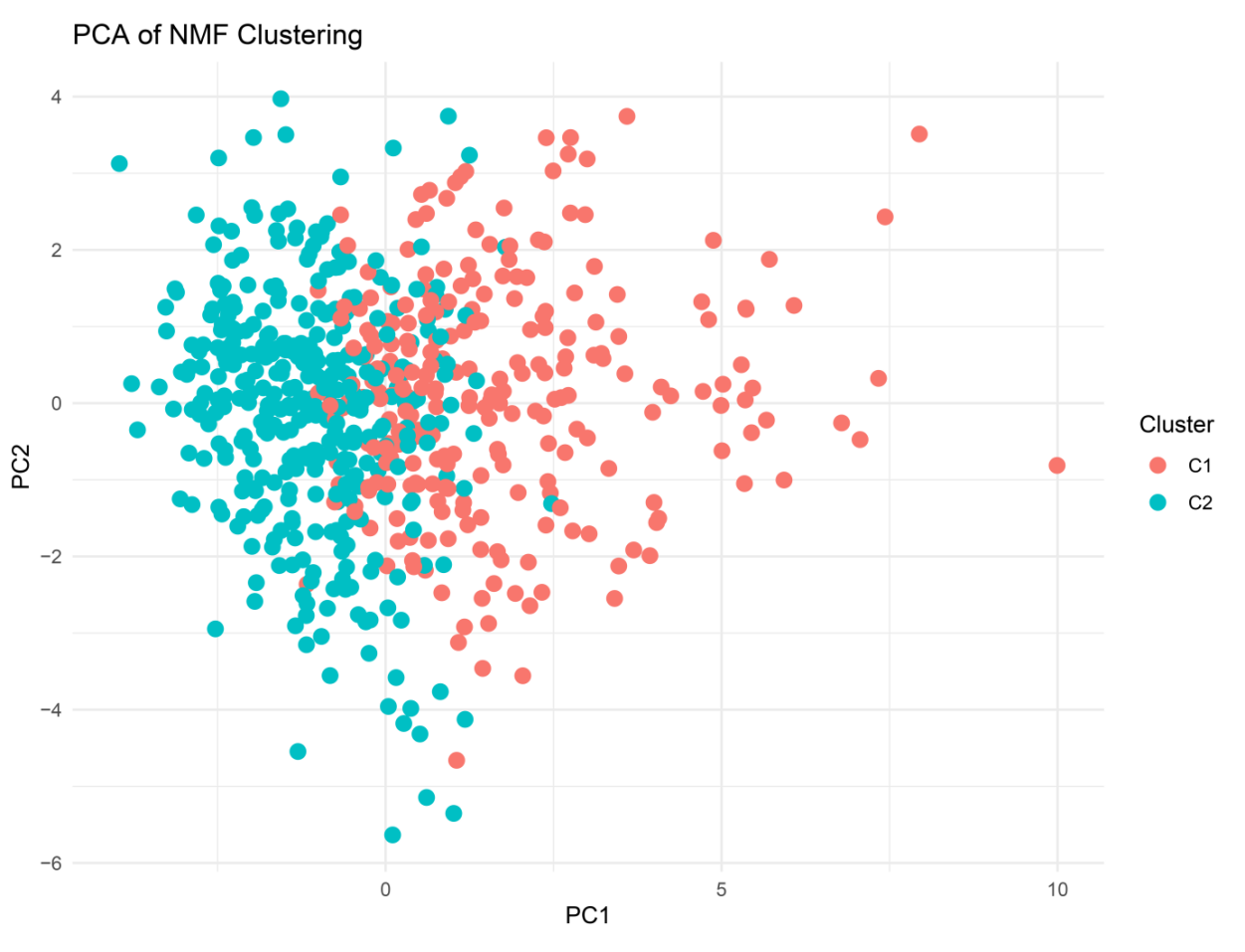**  **Figure S5. Tumor purity and molecular classification features of the NMF-derived CRC subtypes.** (A) Violin plots showing ESTIMATE-derived tumor purity in the two NMF subtypes (C1 and C2). Tumor purity was calculated using the ESTIMATE algorithm; the central line indicates the median and the box denotes the interquartile range. ****P* < 0.001. (B) Distribution of consensus molecular subtypes (CMS1–CMS4) across C1 and C2. CMS classes were inferred using CMScaller based on bulk transcriptomic profiles, and differences in CMS composition between subtypes were evaluated using Fisher’s exact test (*P* = 1×10⁻⁵). (C) Distribution of MSI status across C1 and C2. MSI status was dichotomized as MSI-H (dMMR) versus MSI-L/MSS (pMMR), and differences between subtypes were assessed using Fisher’s exact test (*P* = 0.0954).  **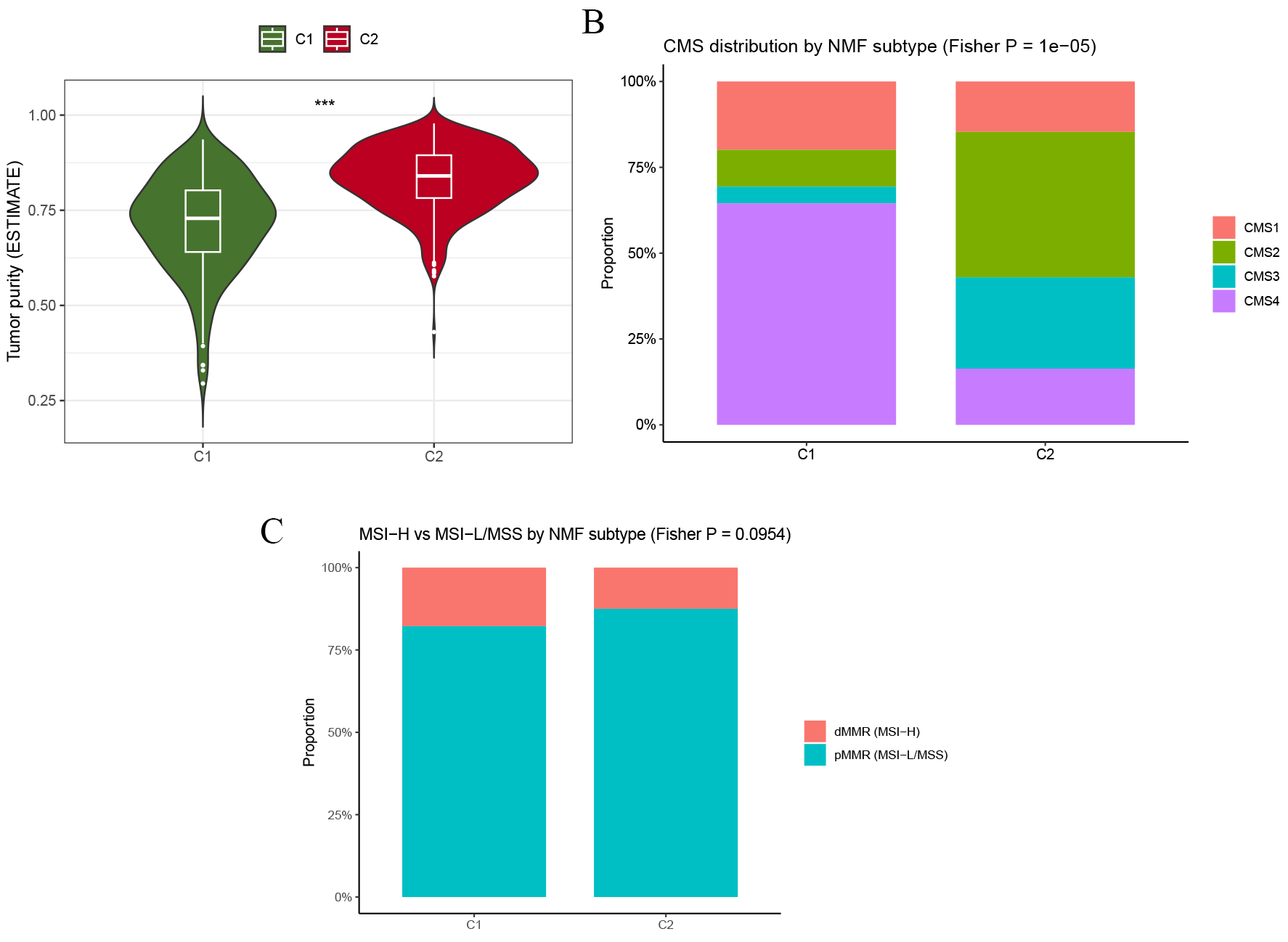**  **Figure S6. Box plot of drug sensitivity analysis between different risk groups.**  **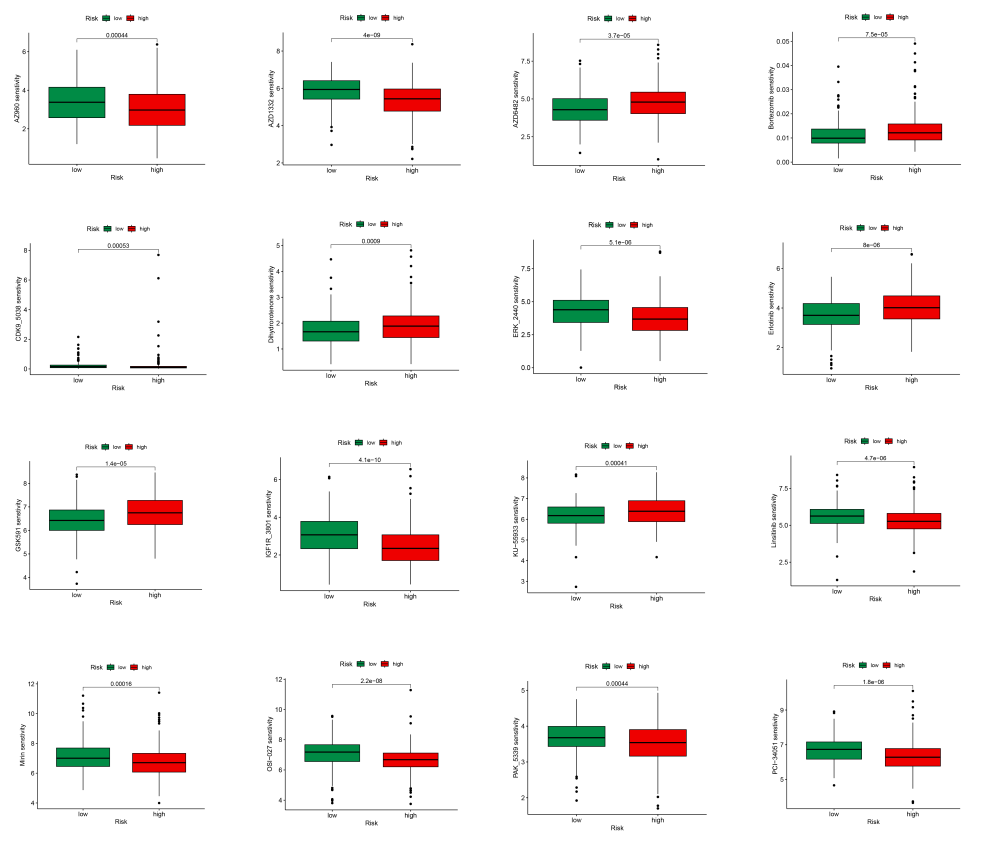**  **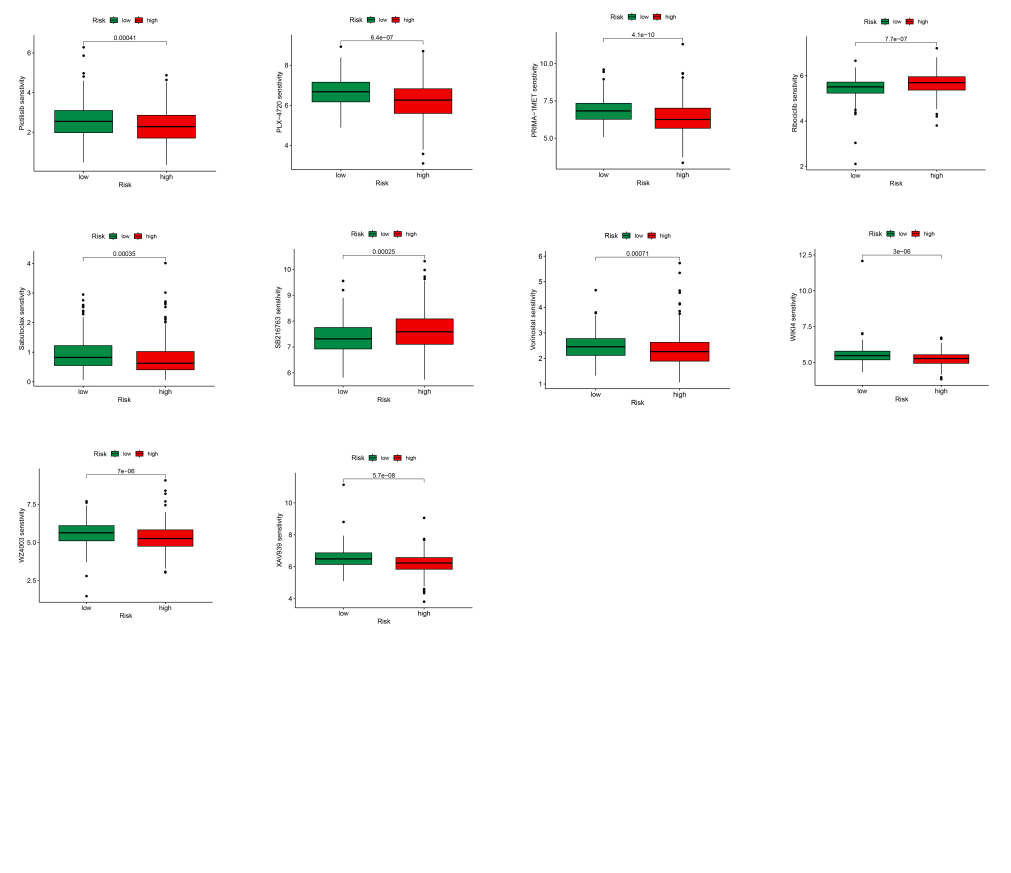** |
| --- |
