# Supplementary material for: Integrative machine learning models reveal immune and metabolic signatures predictive of colorectal cancer prognosis
Source: Discov Oncol. 2026 Mar 3;17:742. doi: 10.1007/s12672-026-04758-y (PMC13187096; doi:10.1007/s12672-026-04758-y)
Supplement: Supplementary file 2 — Supplementary Material 2. [file 12672_2026_4758_MOESM2_ESM.docx]

**Table S3 Antibodies used in Western blot (WB)**

| Primary antibodies | Dilution in WB | Source species | company | Catalog No. |
| --- | --- | --- | --- | --- |
| IL20RB | 1:1000 in WB | Rabbit | Proteintech | 20521-1-AP |
| β-actin | 1:4000 in WB | Rabbit | Proteintech | 20536-1-AP |
| β-Tubulin | 1:8000 in WB | Rabbit | Proteintech | 10094-1-AP |

| Secondary antobody | Dilution |  | company | Catalog No. |
| --- | --- | --- | --- | --- |
| HRP Goat Anti-Rabbit IgG (WB) | 1:4000 |  | Proteintech | SA00001-2 |
