# Supplementary material for: Integrative machine learning models reveal immune and metabolic signatures predictive of colorectal cancer prognosis
Source: Discov Oncol. 2026 Mar 3;17:742. doi: 10.1007/s12672-026-04758-y (PMC13187096; doi:10.1007/s12672-026-04758-y)
Supplement: Supplementary file 3 — Supplementary Material 3. [file 12672_2026_4758_MOESM3_ESM.docx]

**Table S4 Primers sequences used in qRT-PCR**

| Gene | Forward | Reverse |
| --- | --- | --- |
| IL20RB | AGGCCCAGACATTCGTGAAG | CGACCACAAGGATCAGCATGA |
| β-actin | CTCGCCTTTGCCGATCC | GGCTGTTGCATACTTCTCATGG |
