# Supplementary material for: Integrative machine learning models reveal immune and metabolic signatures predictive of colorectal cancer prognosis
Source: Discov Oncol. 2026 Mar 3;17:742. doi: 10.1007/s12672-026-04758-y (PMC13187096; doi:10.1007/s12672-026-04758-y)
Supplement: Supplementary file 5 — Supplementary Material 5. [file 12672_2026_4758_MOESM5_ESM.docx]

**Table S2. Clinicopathological characteristics of patients with colorectal cancer (n = 30).**

| **Characteristic** | **Category** | **n (%)** |
| --- | --- | --- |
| Age (years) | ≤60 | 14 (46.7) |
|  | >60 | 16 (53.3) |
| Sex | Male | 18 (60.0) |
|  | Female | 12 (40.0) |
| Tumor location | Colon | 17 (56.7) |
|  | Rectum | 13 (43.3) |
| Tumor differentiation | Well/Moderately differentiated | 19 (63.3) |
|  | Poorly differentiated | 11 (36.7) |
| T stage | T1–T2 | 12 (40.0) |
|  | T3–T4 | 18 (60.0) |
| N stage | N0 | 16 (53.3) |
|  | N1–N2 | 14 (46.7) |
| M stage | M0 | 27 (90.0) |
|  | M1 | 3 (10.0) |
| TNM stage | I–II | 15 (50.0) |
|  | III–IV | 15 (50.0) |

Data are presented as n (%).
