# Supplementary material for: Integrative machine learning models reveal immune and metabolic signatures predictive of colorectal cancer prognosis
Source: Discov Oncol. 2026 Mar 3;17:742. doi: 10.1007/s12672-026-04758-y (PMC13187096; doi:10.1007/s12672-026-04758-y)
Supplement: Supplementary file 7 — Supplementary Material 7. [file 12672_2026_4758_MOESM7_ESM.docx]

**Marked WB supplementary material**

1. Expression of IL20RB in one type of normal intestinal epithelial cell (NCM460) and four types of colorectal cancer cells

| 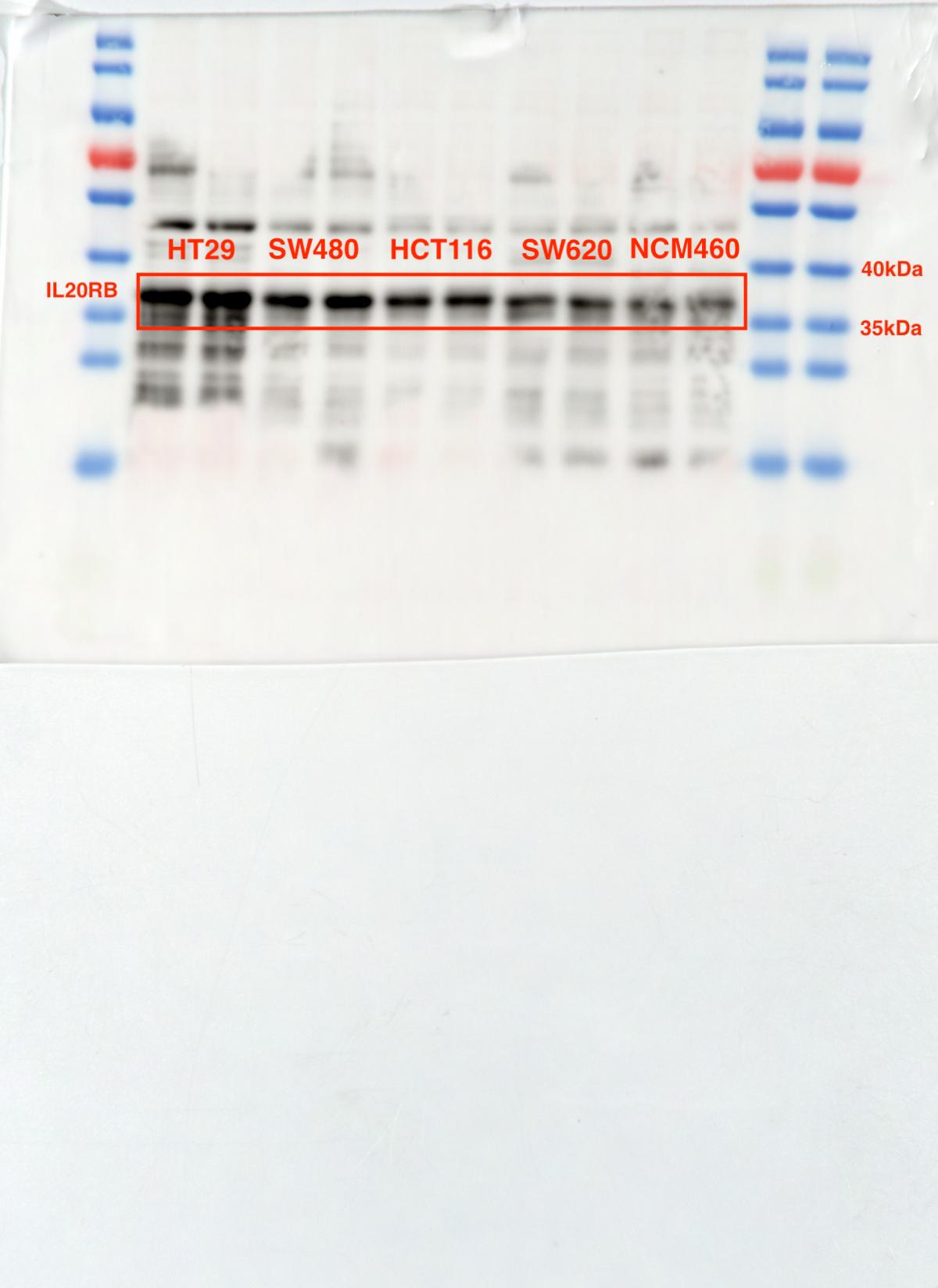  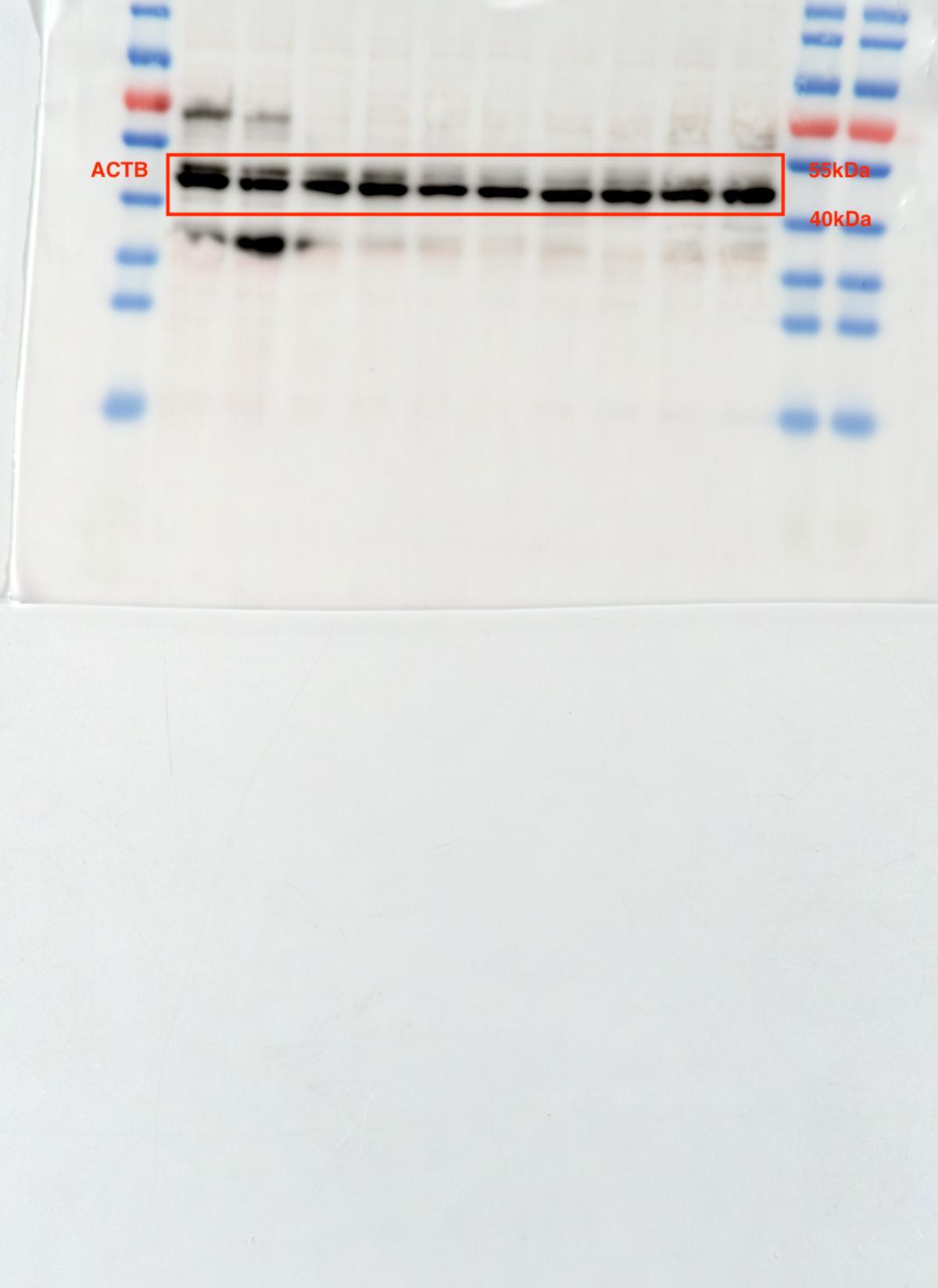 |
| --- |
|  |

2. Expression of IL20RB in normal epithelial tissue and colorectal cancer tissue

| 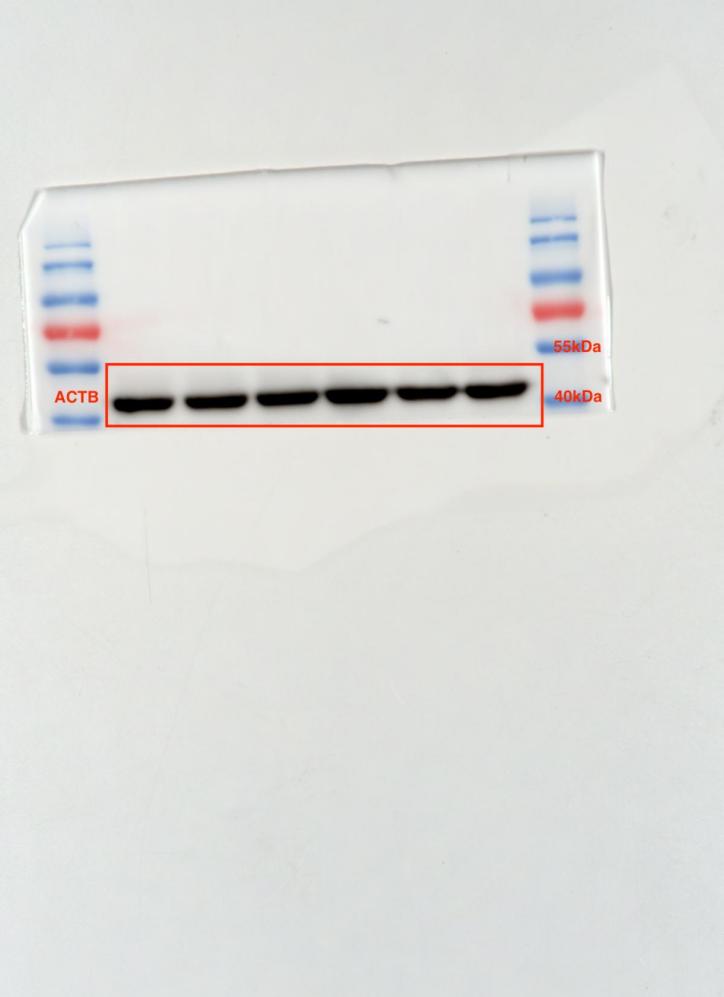 | 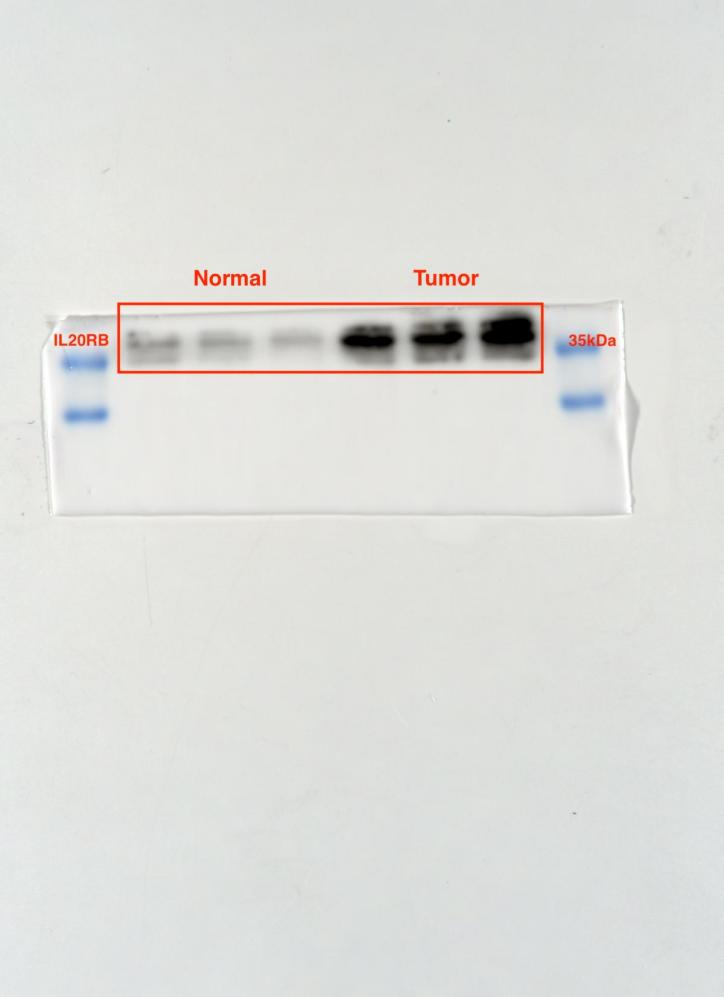 |
| --- | --- |

3. Validation of small interference technique for knocking down IL20RB in HT29 cells

| 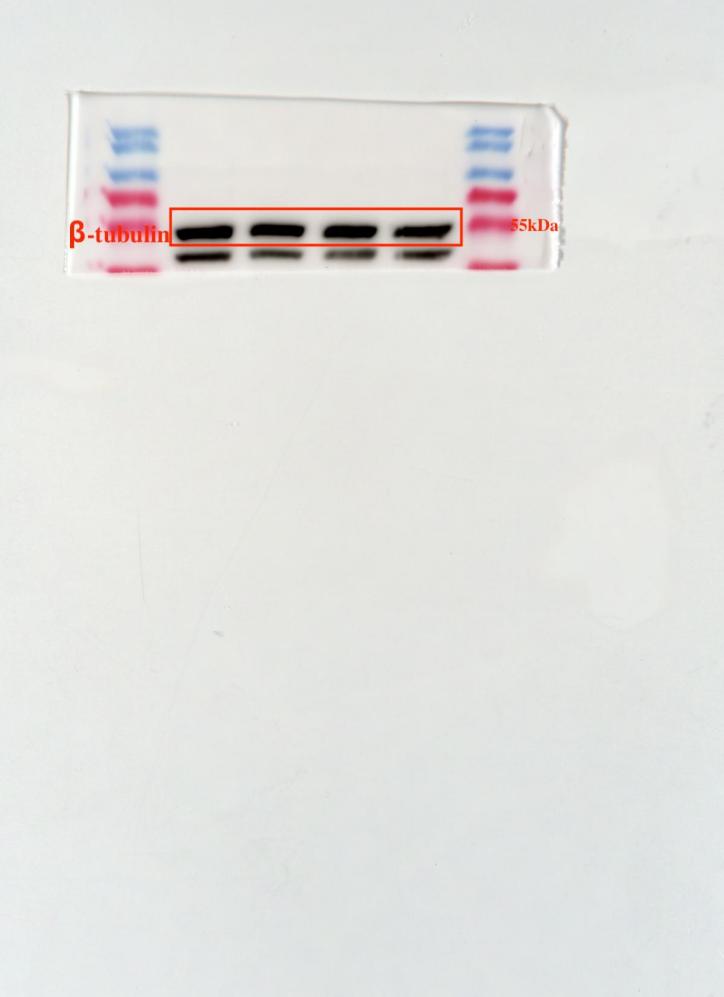 | 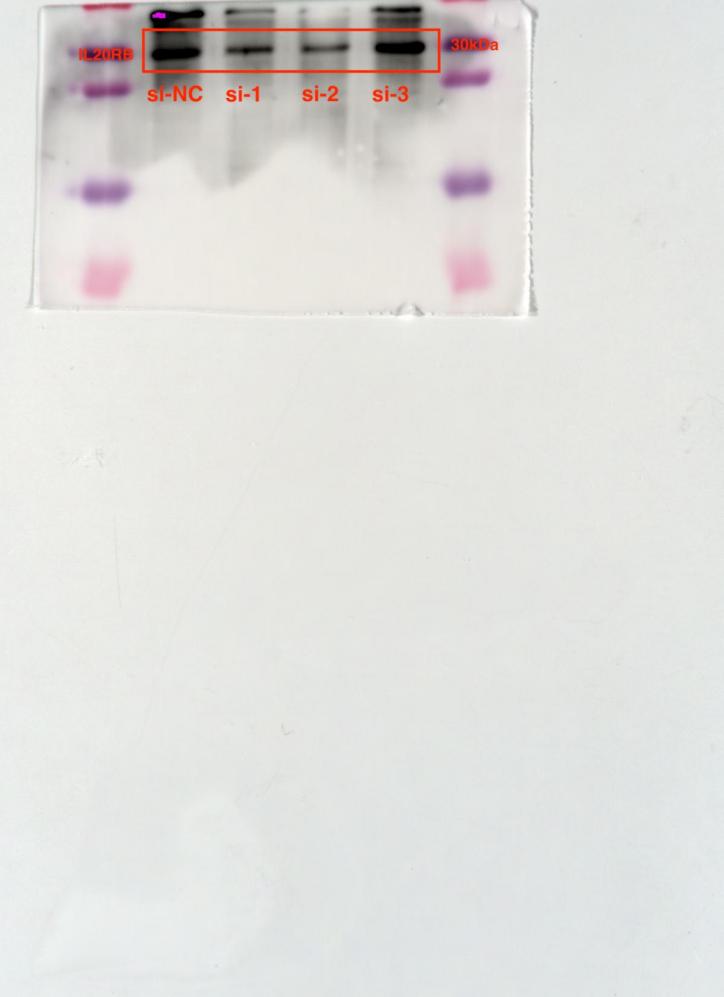 |
| --- | --- |

4. Validation of small interference technique for knocking down IL20RB in SW620 cells

| 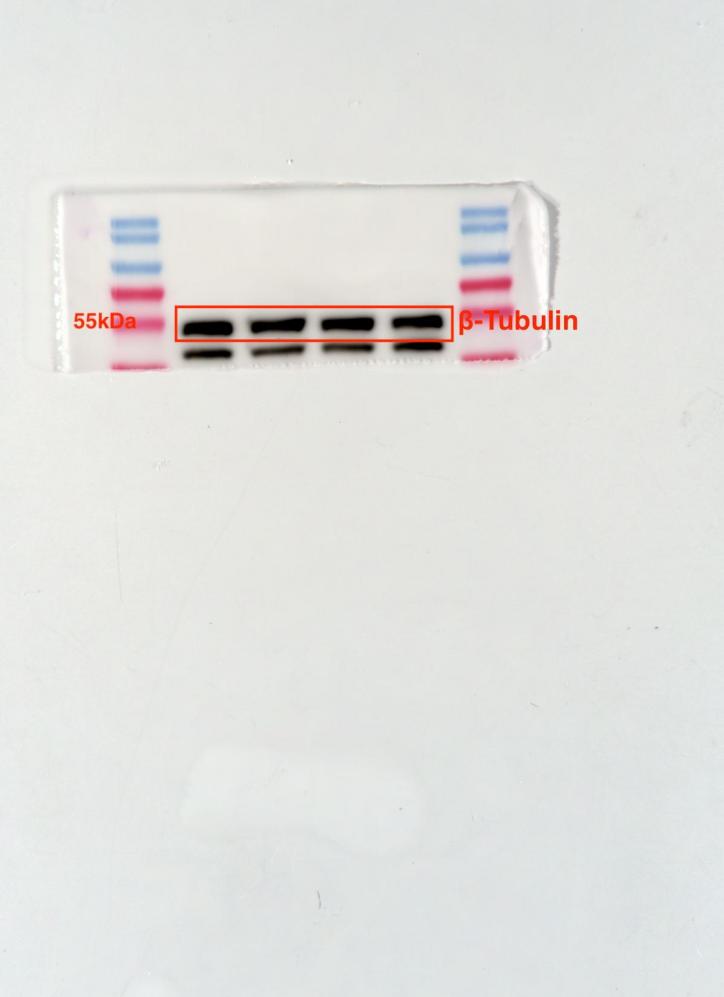 | 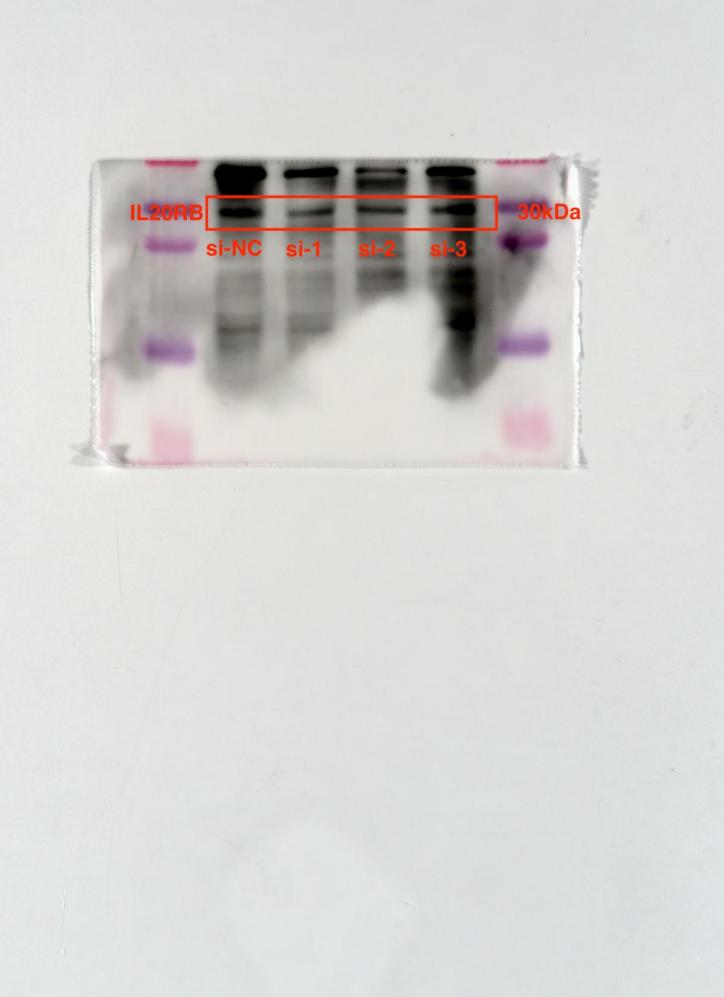 |
| --- | --- |
